# Perpendicular-anisotropy artificial spin ice with spontaneous ordering: a platform for reservoir computing with flexible timescales

**DOI:** 10.1038/s44172-025-00499-y

**Published:** 2025-11-03

**Authors:** Aleksandr Kurenkov, Jonathan Maes, Aleksandra Pac, Gavin Martin Macauley, Bartel Van Waeyenberge, Aleš Hrabec, Laura Jane Heyderman

**Affiliations:** 1 Laboratory for Mesoscopic Systems, Department of Materials, ETH Zurich, Zurich, Switzerland; 2 PSI Center for Neutron and Muon Sciences, Villigen PSI, Switzerland; 3 DyNaMat, Department of Solid State Sciences, Ghent University, Ghent, Belgium

**Keywords:** Magnetic properties and materials, Magnetic properties and materials, Computational science

## Abstract

Arrays of coupled nanomagnets have wide-ranging fundamental and practical applications in artificial spin ices, reservoir computing and spintronics. However, lacking in these fields are nanomagnets with perpendicular magnetic anisotropy with sufficient magnetostatic interaction. This would not only open up unexplored possibilities for artificial spin ice geometries but also enable novel coupling methods for applications. Here, we demonstrate a method to engineer the energy landscape of artificial spin lattices with perpendicular magnetic anisotropy. With this, we are able to realize for the first time strongly magnetostatically-coupled 2D lattices of out-of-plane Ising spins that spontaneously order at room temperature on timescales that can be precisely engineered. We show how this property, together with straightforward electrical interfacing, make this system a promising platform for reservoir computing. Our results open the way to investigate the thermodynamics of out-of-plane magnetostatically coupled nanomagnet arrays with novel spin ice geometries, as well as to exploit such nanomagnet arrays in unconventional computing, taking advantage of the adjustable temporal dynamics and strong coupling between nanomagnets.

## Abstract (web summary)

Aleksandr Kurenkov and colleagues demonstrate nanomagnet arrays with out-of-plane anisotropy that spontaneously order at room temperature. This system offers a new platform for unconventional computing through tunable dynamics.

## Introduction

Coupled nanomagnets organized on the sites of various lattices are widely-explored in the field of artificial spin ice^1–3^ since they exhibit a large variety of fascinating phenomena including collective dynamics^4–6^, frustration^7–9^, dynamic chirality^10^ and phase transitions^11–13^. Furthermore, these properties can be exploited for novel forms of conventional^14–19^ and unconventional^20–24^ computing. The single-domain nanomagnets typically have an in-plane magnetic anisotropy, which results in strong magnetostatic coupling between the magnets due to extended demagnetizing fields in the lattice plane. In contrast, dipolar-coupled nanomagnets with perpendicular magnetic anisotropy have rarely been employed for artificial spin ice because they have not shown spontaneous ordering–so they are not “thermally-active”–at experimentally measurable timescales^25–30^. This lack of spontaneous evolution makes such magnets a common choice for high-density bit-patterned media^31,32^.

Creating lattices comprised of magnetostatically coupled nanomagnets with perpendicular anisotropy that show spontaneous ordering on an experimentally-accessible timescale would open the way to study the thermodynamics of out-of-plane artificial spin ices based on a large variety of lattices. Among them are the lattices associated with the crystal planes of many bulk magnetic systems^33–35^, the triangular lattice^36,37^, and the canonical two-dimensional (2D) Ising model on the square lattice^38–40^. While any of these lattices can be created with the method described in this work, we will focus on the latter due to its broad importance and universality. Being universally complete, so that any other statistical model^41,42^ or Boolean circuit^43,44^ can be derived from it, the 2D Ising model is of fundamental importance for statistical physics and has been used to model numerous physical, mathematical and biological processes^45–47^. The modelling is typically carried out by selecting a particular variant of the model, setting an initial state, and then observing how it evolves into a lower-energy state over time. The nature of this evolution provides a means to solve non-deterministic polynomial-time (NP-) hard problems with only polynomial overhead (P-hard) by mapping them onto a corresponding Ising system^48,49^. Since any NP-hard problem can be formulated as an Ising problem, the 2D Ising system is of great interest for a variety of combinatorial problems^46^. In addition, energy minimization and state dynamics in 2D Ising lattices bear similarities to equivalent processes in the human brain^50^, providing a basis for computational models of the brain^50,51^ and several types of neural networks such as Boltzmann machines^52^ or Hopfield networks^53^.

**Fig. 1:**
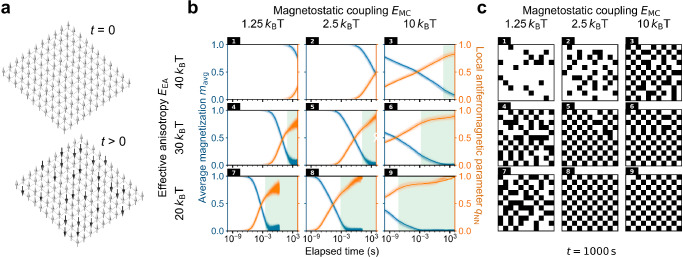
Dependence of the ordering timescale on the energy balance in a 2D Ising square lattice. **a** Schematic of the system with 11 × 11 spins. It is initialized to a uniform magnetic state with all spins pointing up and then released at *t* = 0. The lattice then relaxes to lower energies for *t* > 0. This system size was chosen because it matched the size of our fabricated systems discussed later. **b** Evolution of the local antiferromagnetic parameter *q*_NN_ and the average magnetization *m*_avg_ for different effective anisotropy *E*_EA_ and magnetostatic coupling *E*_MC_. 20 simulations (fine lines) and their average (bold line) are shown for each case. The green-shaded region highlights the second stage of ordering where the slope of *q*_NN_ decreases and involves the reversal of spins within the domain boundaries. The simulations are stopped after 1000 s and the resulting magnetic states are shown in **c**. **c** Magnetic states at *t* = 1000 s. Here white (black) contrast corresponds to the up (down) spins. Panels 1–9 correspond to those of **b**.

Here, we create for the first time a lattice of magnets with out-of-plane anisotropy that spontaneously orders at room temperature. We focus on a 2D square lattice of Ising spins (Fig. 1a) because of its importance for fundamental science and for applications. By fabricating such lattices with nanomagnets with various diameters and separations, and made from precisely tuned multilayer films with different number and thicknesses of the layers, we have been able to determine both theoretically and experimentally how the energy landscape of the system defines its ordering dynamics. Utilizing this information, we have engineered the timescale of the system response, which provides a means to tailor the arrays for specific computing applications.

## Results

### Relating the energy landscape to the magnetic relaxation timescale with Monte Carlo simulations

We begin with a theoretical exploration of the degree and timescale of spontaneous ordering in a square 2D Ising system and how it is influenced by the energy landscape of the spins. For this, we performed kinetic Monte Carlo simulations using the “Hotspice” package^54^. The system simulated was a lattice of 11 × 11 magnetostatically (dipolarly, in this case) coupled Ising spins (Fig. 1a) in contact with a heat bath of temperature *T*. The lattice was first initialized in a state with all spins pointing up (upper panel of Fig. 1a) and allowed to relax for 1000 seconds. This time was chosen to match the approximate time between the initialization and measurement of the experimental system discussed later, with the average magnetization *m*_avg_ and local antiferromagnetic parameter *q*_NN_ tracked during this period. We define *q*_NN_ as $(1-\langle {S}_{i}{S}_{i+1}\rangle )/2$, where $\langle {S}_{i}{S}_{i+1}\rangle$ is the nearest-neighbour correlation, and the mean switching time of a spin *j* at a time *t* is given by the Néel-Arrhenius law^55–57^:

1 $${\tau }_{j}(t)={\tau }_{0} \exp \left(\frac{\Delta {E}_{j}(t)}{{k}_{B}T}\right)$$

Here *t* is elapsed time, ${\tau }_{0}={10}^{-10}{{\rm{s}}}$ is the attempt period and $\Delta {E}_{j}(t)$ is the energy barrier

2 $$\Delta {E}_{{\mbox{j}}}\left(t\right)={E}_{{{\rm{EA}}}}\left(1+{\mathrm{rand}}\left(j\right)\right)+{E}_{{{\rm{MC}}}}{\sum}_{i,j}{S}_{i}{\left(t\right){D}_{{ij}}S}_{j}(t),$$

defined by a configuration of spins ${S}_{i}$ at every time step, magnitude of the point dipolar interaction ${D}_{{ij}}$ between spins *i* and *j*, a random ±5% Gaussian variation of anisotropy ${\mathrm{rand}}(j)$ for different spins^24,58^, as well as a time-independent effective anisotropy energy *E*_EA_ and energy associated with the dipolar coupling between the nearest neighbour spins *E*_MC_ (see Supplementary Note 1 for details).

The initial switching of individual nanomagnets is promoted by dipolar interactions with other nanomagnets in the lattice given by ${E}_{{{\rm{MC}}}}{\sum}_{i,j}{S}_{i}{\left(t\right){D}_{{ij}}S}_{j}(t)$ but, as the system assumes a more ordered state, this term decreases in value. This results in an exponential increase of the switching time due to the exponential term in Eq. 1. Simulations carried out for several sets of *E*_EA_ and *E*_MC_ confirm the universally logarithmic dependence of *m*_avg_ and *q*_NN_ on the elapsed time (Fig. 1b). An increase in *E*_MC_ (see columns in Fig. 1b) extends the relaxation dynamics over a longer timescale, while changing *E*_EA_ (see rows in Fig. 1b) does not change the slope of the curves and only shifts them along the time axis. Irrespective of the *E*_EA_ and *E*_MC_, the spin lattices achieve *m*_avg_ ~ 0 (blue traces in Fig. 1b) earlier than perfect checkerboard ordering with *q*_NN_ = 1 (orange traces in Fig. 1b). Furthermore, as the system nears *m*_avg_ ~ 0, the ordering rate decreases, which can be seen by the decrease in the slope of the orange lines as they enter the green-shaded regions in Fig. 1b. Snapshots of the magnetic state at *t* = 1000 s (Fig. 1c) provide a clue to why this two-stage ordering process occurs (reflected by the two different slopes in *q*_NN_). Panels 1 and 2 in Fig. 1c are snapshots of the system during the rapid change of both *m*_avg_ and *q*_NN_ in the first phase of the ordering. Here, individual nanomagnets throughout the system switch (Panel 1) followed by domains of antiferromagnetic ordering starting to form (Panel 2). These doubly degenerate domains expand to completely fill the system (Panel 3 and 4) with boundaries forming between the domains with nanomagnets of opposite polarity (Panels 5 and 6).

From this point on, a further increase in *q*_NN_ can only be achieved by switching the nanomagnets in the domain boundaries. As these magnets are now stabilized through the dipolar interactions with their neighbours that have already switched, the energy barrier for them to switch is considerably higher than at the beginning of the relaxation process. This increases the switching time exponentially (see Eq. 1) and slows down further ordering. The details of this process are elaborated in Supplementary Note 2. A consequence of the two-stage ordering is that, while a stronger coupling *E*_MC_ promotes faster demagnetization *m*_avg_ → 0 (e.g., see blue lines in Panels 7–9 of Fig. 1b, where the time to get to *m*_avg_ ~ 0 decreases from ~10^-2^ to ~10^-3^), it does not necessarily result in faster ordering *q*_NN_ → 1 (orange lines in Panels 7–9 of Fig. 1b) as it increases the stabilization of the magnetic states in the domain boundaries. Therefore, *E*_MC_ must not exceed a certain upper limit for the system to order on a given timescale and to not dwell in a metastable state with *m*_avg_ ~ 0 and *q*_NN_ < 1.

**Fig. 2:**
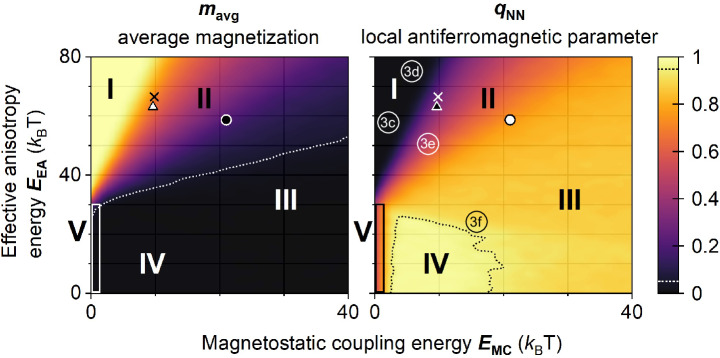
Phase diagrams of the average magnetization *m*_avg_ and the local antiferromagnetic parameter *q*_NN_ as a function of effective anisotropy *E*_EA_ and magnetostatic coupling *E*_MC_ at *t* = 1000 s. Five regions can be distinguished: I – frozen state, II – transient states, III – state with domains and domain boundaries, IV – checkerboard ordering, V – superparamagnetic state. The white (black) dotted line corresponds to *m*_avg_ = 0.05 (*q*_NN_ = 0.95). Labels 3c-3f correspond to the energy landscapes shown in Fig. 3c–f. The three symbols correspond to the experimental lattices with nanomagnet diameter *D*_NM_ = 170 nm, Co thickness *t*_Co_ = 1.45 nm and nanomagnet separation *S*_ASI_ = 20 nm (circle), *S*_ASI_ = 25 nm (triangle), *S*_ASI_ = 30 nm (cross). The experimental magnetic configurations of these systems are shown in Fig. 4.

The dependence of *m*_avg_ and *q*_NN_ on *E*_EA_ and *E*_MC_ at *t* = 1000 s is summarized in the phase diagrams of Fig. 2. In Region I, neither *m*_avg_ nor *q*_NN_ experience a notable change compared to the initial state due to the strong anisotropy and weak magnetostatic coupling (corresponding to Panel 1 in Fig. 1c). Increase of the *E*_MC_/*E*_EA_ ratio shifts the system through the transient Region II, characterised by decreasing average magnetization and increasing ordering (corresponding to Panel 2 in Fig. 1c). Further increase of *E*_MC_/*E*_EA_ shifts the system to Region III with *m*_avg_ ~ 0 and *q*_NN_ still not 1, characteristic for the second ordering stage (corresponding to Panel 6 of Fig. 1c). Any further increase of *E*_MC_/*E*_EA_ does not affect the average magnetization or ordering at *t* = 1000 s. Achieving the high ordering of Region IV, given by the dotted black line that envelops a region with *q*_NN_ ≥ 0.95 in Fig. 2 (exemplified by Panels 8 and 9 in Fig. 1c), requires lowering both *E*_MC_ and *E*_EA_ below a certain threshold. The narrow Region V (enclosed by vertical box in Fig. 2) is the regime in which the spins are superparamagnetic at the chosen timescale. The border between Regions V and I at *E*_MC_ = 0 is therefore a transition between the superparamagnetic and frozen regimes. This border is located at *E*_EA_ ~ 30 *k*_B_*T* because nanomagnets with such an effective anisotropy have an average switching time of ~1000 s (the time point of these diagrams). Region IV with *q*_NN_ ~ 1 is, therefore, constrained from the left by the superparamagnetic regime, from the top by the frozen regime, and from the right by states in which switching spins in domain boundaries is too energetically expensive.

The phase diagrams in Fig. 2 are snapshots at *t* = 1000 s and, with increasing time, the boundaries between the different regions will shift. Because of this, if the observation time is long enough and *E*_MC_ is non-negligible, a system located in the frozen region at *t* = 1000 s may eventually find itself in the more ordered Regions II, III or IV (see phase diagrams for *t* ~ 27 months in Supplementary Note 3). The state of the nanomagnet array is therefore defined by *E*_EA_, *E*_MC_ and *t*. Practical applications exploiting systems with specific dynamics defined by *E*_EA_ and *E*_MC_ require careful adjustment of the lattice and nanomagnet dimensions, as well as the stack materials and layer thicknesses. We show how this can be achieved in the next section.

### Designing the energy landscape of coupled Co/Pt nanomagnets

**Fig. 3:**
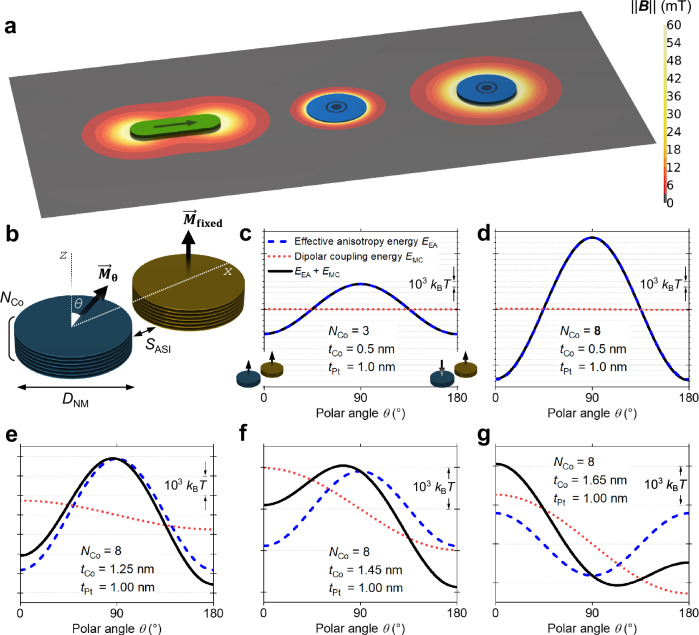
Dependence of the energy landscape of multilayered nanomagnets with perpendicular magnetic anisotropy on the material stack and lateral dimensions. **a** Magnitude of the magnetic flux density for a permalloy nanomagnet (dimensions: 100 × 300 × 20 nm^3^) with in-plane anisotropy (left) and 200 nm-diameter nanomagnets with perpendicular magnetic anisotropy consisting of 3 Co layers of 0.5 nm thickness (centre) and 8 Co layers of 1.45 nm thickness (right). The Co layers are separated by 0.8 nm of vacuum. **b** Schematic showing the geometrical parameters used in energy landscape calculations. **c**–**g** Energy landscapes calculated for the system shown in **b** for *D*_NM_ = 200 nm, *S*_ASI_ = 20 nm and different Co layer thicknesses. The vertical scale is energy, where *k*_B_ is the Boltzmann constant and *T *= 300 K.

Artificial spin ices with perpendicular magnetic anisotropy that are thermally-active at room temperature on experimentally measurable timescales have not been realized before. The first reason for this is that achieving large *E*_MC_ for this geometry is harder due to the confinement of demagnetizing fields in the vicinity of the nanomagnet (central magnet in Fig. 3a) compared to the in-plane case (leftmost magnet in Fig. 3a). The second reason is that balancing the energy contributions to *E*_EA_, required to lower the energy barrier to switching, is equally challenging in Co/x (x = Pt, Pd, Ni) multilayers. To address these challenges, we focused on precisely controlling *E*_EA_ in perpendicular nanomagnets and maximizing *E*_MC_ between them.

We fabricated the nanomagnets from Co/Pt ferromagnetic multilayers, which are widely used because of their strong perpendicular magnetic anisotropy^59^. The primarily interfacial origin of the anisotropy allowed us to vary *E*_EA_ and *E*_MC_ almost independently by changing the number of Co/Pt interfaces and the thickness of the Co layers, respectively. We have calculated the energy landscape of a pair of nanomagnets to guide the selection of nanomagnet diameter *D*_NM_, separation *S*_ASI_, number of Co layer repetitions *N*_Co_, and thicknesses of Co and Pt layers, *t*_Co_ and *t*_Pt_, respectively (Fig. 3b). We allowed the magnetization to rotate coherently in one nanomagnet (${\vec{M}}_{{{\rm{\theta }}}}$ in Fig. 3b) while keeping the other fixed (${\vec{M}}_{{{\rm{fixed}}}}$ in Fig. 3b). The energy was then calculated for polar angles *θ* from 0° to 180° and included four energy terms associated with the uniaxial interfacial anisotropy, demagnetization, magnetostatic interaction between the layers in the nanomagnet and the dipolar coupling between the nanomagnets (see Supplementary Note 4 for details). The resulting energy landscape provides an estimate of the energy barrier that the system needs to overcome to go from the higher-energy magnetic state with parallel moments to the lower-energy antiparallel state.

In Fig. 3c, we show the energy landscape for a pair of coupled nanomagnets made of (Co [0.5] / Pt [1.0])_2_ / Co [0.5] layers with *N*_Co_ = 3, *D*_NM_ = 200 nm and *S*_ASI_ = 20 nm, where the numbers in square brackets are thicknesses in nm. Such stacks have a high anisotropy and are widely used for spintronics applications^60,61^ due to the high thermal stability, which provides more than 10 years of retention time (a measure of how long a device can store information reliably without the nanomagnet switching) even in sub-20 nm diameter nanomagnets^60^. The small number of ferromagnetic Co layers, as well as their low thickness, results in a localized demagnetizing field (central magnet in Fig. 3a), minimizing crosstalk between the nanomagnets. Both properties–high anisotropy and low crosstalk–while useful for information storage applications, contradict the high-*E*_MC_, low-*E*_EA_ requirements of a lattice to give fast spontaneous ordering. Arrays of nanomagnets made of such a stack are located in the “frozen” Region I of Fig. 2 (“3c” label).

To devise a high-*E*_MC_, low-*E*_EA_ stack, we varied *N*_Co_ and *t*_Co_ while fixing the nanomagnet diameter *D*_NM_ and separation *S*_ASI_. *S*_ASI_ should be minimized to maximize the dipolar coupling, and was set to 20 nm, which was the smallest nanomagnet separation we were able to obtain with confidence when fabricating nanomagnet arrays with electron beam lithography. *D*_NM_ was set to 200 nm, which was the largest possible nanomagnet diameter that did not result in the formation of multidomain states. This upper threshold value for *D*_NM_ was determined experimentally by observing the magnetic states in multiple lattices of nanomagnets fabricated with different diameters.

Increasing the number of Co layer repetitions *N*_Co_ from 3 to 8 produces a stack similar to those used in previous works on artificial spin ices with out-of-plane nanomagnets^25–29^, with the energy landscape shown in Fig. 3d. *E*_MC_ is approximately quadratically proportional to the total Co thickness and increases by a factor of ~7. However, the increased number of Co/Pt interfaces results in a higher anisotropy, as seen in Fig. 3c, d, and the system remains in Region I of Fig. 2 (“3d” label). Achieving an *E*_MC_-dominated lattice requires the lowering of *E*_EA_ without decreasing *E*_MC_. For this, one needs to consider the two additional components of *E*_EA_ beyond the interfacial anisotropy: the demagnetization energy and dipolar coupling between layers of the stack. The dipolar coupling between the layers is rather weak and increases the perpendicular anisotropy, and thus cannot be used to lower *E*_EA_. In contrast, the demagnetization energy is considerable and decreases *E*_EA_. The demagnetization energy increases with Co thickness, just like *E*_MC_, which has the same physical origin. Therefore, increasing *t*_Co_ to 1.25 nm results in a reduction in *E*_EA_ and an increase in *E*_MC_ (Fig. 3e), shifting the system towards Region II of Fig. 2 (“3e” label). At *t*_Co_ = 1.45 nm (Fig. 3f), *E*_MC_ ~ *E*_EA_, which results in a highly asymmetric landscape with the lowest *E*_EA_ and highest *E*_MC_ among the considered stacks. Such an energy landscape should facilitate spontaneous ordering and, depending on the absolute values of *E*_EA_ and *E*_MC_, an array of such nanomagnets belongs to Region III or IV of Fig. 2 (“3f” label). The demagnetizing field of these nanomagnets (rightmost nanomagnet in Fig. 3a) has a similar extent to that of the Permalloy nanomagnets typically implemented for in-plane artificial spin ices (leftmost nanomagnet in Fig. 3a). Further increase of *t*_Co_ to 1.65 nm results in the shift of the energy minimum to *θ* ~ 110°, indicating a loss of perpendicular anisotropy (Fig. 3g).

From calculations, we have shown the effect on the energy landscape of changing the experimental system in terms of layer thicknesses, as well as the nanomagnet diameters and separations. By comparing this to the results from our Monte Carlo simulations, we are then able to predict how changing the system parameters will influence the relaxation timescale. This has enabled us to experimentally realize square lattices of interacting out-of-plane nanomagnets with the ability to control the ordering timescales from sub-seconds to years, as we show in the following section.

### Control of relaxation timescales in experimental dipolar-coupled 2D Ising systems

We now turn to experimental systems where limitations in fabrication impose additional constraints on the system design. For example, achieving small *S*_ASI_—a key parameter influencing *E*_MC_—becomes more challenging as the thickness of the stack increases, so we limit *N*_Co_ to 7. Another effect is that the Pt spacer layers may become discontinuous on decreasing their thickness below ∼0.4 nm and, to be sure that we have a continuous layer we choose *t*_Pt_ = 0.8 nm for the experimental systems, unless otherwise mentioned. These parameters permitted an *S*_ASI_ down to 20 nm, and we varied *S*_ASI_, *D*_NM_ and *t*_Co_ in the experiment.

The multilayers of Ta[3] / Pt[4] / (Co[*t*_Co_] / Pt[0.8])_6_ / Co[*t*_Co_] / Ru[2] were deposited by magnetron sputtering on Si substrates with a natural oxide layer. They were patterned into square lattices of 11×11 nanomagnets by electron beam lithography and Ar ion milling. For transport measurements, the Ta/Pt bottom layer was patterned into Hall bars. Magnetic force microscopy (MFM) was used to measure the magnetic state after applying an out-of-plane magnetic field of 0.9 T to initialize all nanomagnets to an “up state”. For the MFM measurements, we covered the samples with a 60 nm-thick Poly(methyl methacrylate) resist to minimize the influence of the stray magnetic field of the MFM probe on the sample.

**Fig. 4:**
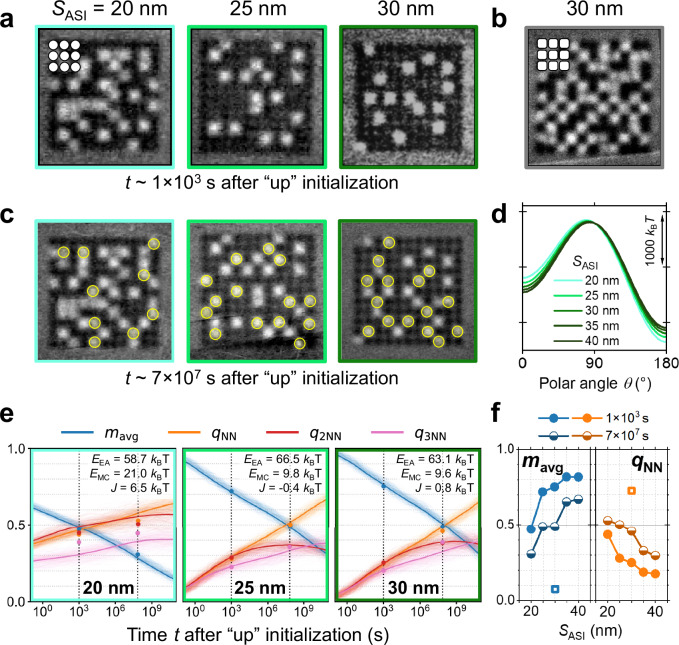
Observing the dependence of the ordering timescale on nanomagnet separation and nanomagnet shape with MFM. All systems were initialized to a uniform magnetic state with an out-of-plane magnetic field before the measurements. **a** MFM images of lattices with *t*_Co_ = 1.45 nm, *D*_NM_ = 170 nm and increasing nanomagnet separation *S*_ASI_ observed at *t* ~ 1000 s after initialization. Black nanomagnets are in the initial magnetic state while white nanomagnets have switched. Image frame colours match those of the corresponding curves in **d** and frames in **e**. **b** Array of 170 nm-wide square nanomagnets separated by 30 nm observed at *t* ~ 1000 s. *t*_Co_ = 1.45 nm as in **a**. The nanomagnet shape is shown in the top left inset. All other results are for circular nanomagnets as shown in the top left inset of **a**. **c** The same nanomagnet arrays as in **a** observed after ~27 months at room temperature. The magnets that have switched during this time are highlighted with yellow circles. **d** Energy landscapes calculated for the lattices shown in **a**. **e** Evolution of average magnetization *m*_avg_ and local antiferromagnetic parameters *q*_NN_, *q*_2NN_, *q*_3NN_. The fine lines are calculated using Monte Carlo simulations, the bold lines are their averages, and the points are experimental data. **f** Average magnetization *m*_avg_ and local antiferromagnetic parameter *q*_NN_ as a function of *S*_ASI_ determined from the MFM images. Data for **a**–**c** are represented as filled circles, open squares and semi-filled circles, respectively. MFM images for *S*_ASI_ = 35 and 40 nm can be found in Supplementary Note 5.

We first looked at arrays of nanomagnets with *t*_Co_ = 1.45 nm, *D*_NM_ = 170 nm and different *S*_ASI_. The state of the arrays at time *t* ~ 1000 s after initialization is shown in Fig. 4a. Black contrast indicates that the nanomagnets are in the state initialized by magnetic field, while white contrast indicates nanomagnets that have spontaneously switched. The number of switched nanomagnets gradually decreases with increase in *S*_ASI_. Since *S*_ASI_ has no effect on *E*_EA_ and only changes *E*_MC_, this result is easy to interpret. The decrease of *E*_MC_ at a constant *E*_EA_ lowers the asymmetry of the energy landscape by making the initial energy well deeper and therefore harder to leave (*θ* = 0° in Fig. 4d) while making the lower-energy well shallower (*θ* = 180° in Fig. 4d).

From the MFM images, we calculated the average magnetization *m*_avg_ and local antiferromagnetic parameter *q*_NN_ as defined above. With decreasing *S*_ASI_, the monotonic increase in *q*_NN_ and decrease in *m*_avg_ (filled circles in Fig. 4f; MFM images for 35 and 40 nm are shown in Supplementary Note 5) highlight the important role that the magnetostatic coupling plays in the spontaneous ordering. We do not reach *q*_NN_ = 1, *m*_avg_ = 0, characteristic of highly ordered states since a further decrease of *S*_ASI_ is challenging in terms of the fabrication. Instead, we changed the shape of the nanomagnets from circular to square with rounded corners (compare insets in Fig. 4a, b) without modifying the stack. The resulting closer proximity of the magnets produces higher *E*_MC_ and a substantial increase in ordering (MFM image in Fig. 4b; *m*_avg_ and *q*_NN_ shown as a square data point in Fig. 4f).

The *m*_avg_ and *q*_NN_ of a 2D Ising system with any *E*_EA_ and *E*_MC_ will evolve with time (see, for example, Fig. 1b). Therefore, one can achieve a higher degree of ordering simply by waiting long enough. To confirm this, we kept the nanomagnet arrays at room temperature in ambient atmosphere and no magnetic field for ~27 months, which is equivalent to ~7 × 10^7 ^s. We then remeasured the samples without applying any magnetic field and observed the states shown in Fig. 4c (additional intermediate magnetic states are shown in Supplementary Note 5). The nanomagnets that have additionally switched are highlighted by yellow circles. There was no switching of nanomagnets from white to black (i.e. back to the state initialized 27 months prior). The new *m*_avg_ and *q*_NN_ are shown in Fig. 4f with half-filled circles. The decrease in *m*_avg_ and increase in *q*_NN_ indicate the additional ordering of the lattice, and one can make use of the evolution of these parameters to pinpoint location of the lattices on the phase diagrams of Fig. 2. For this, we calculated *m*_avg_, *q*_NN_ as well as local antiferromagnetic parameters *q*_2NN_ and *q*_3NN_ for the 2^nd^ and 3^rd^ nearest neighbours from the MFM images for *t* = 1000 s and *t* = 7 × 10^7 ^s. We then fitted these values with *m*_avg_(*t*), *q*_NN_(*t*), *q*_2NN_(*t*) and *q*_3NN_(*t*) calculated using Monte Carlo simulations with the “Hotspice” package^54^, and using *E*_EA_, *E*_MC_ and the ferromagnetic exchange coupling between the magnets *J* as variables (see Supplementary Note 5 for details). The fitted results are shown in Fig. 4e. The non-zero *J* in the case of *S*_ASI_ = 20 nm (*J* ~ 6.5 mT) may indicate incomplete separation of the nanomagnets in the bottom Co layer. The near-zero *J* for *S*_ASI_ = 25 and 30 nm suggests that the magnets are fully separated. The location of the fitted results on the phase diagrams of Fig. 2 (circles, triangles and crosses for *S*_ASI_ = 20, 25 and 30 nm, respectively) indicates that the achieved levels of *E*_MC_ are sufficient for a complete ordering (Region V) in *t* ~ 1000 s but *E*_EA_ is too high. Note that these phase diagrams were calculated assuming that there is no exchange coupling between the nanomagnets, so *J* = 0. Therefore, since the *S*_ASI_ = 20 nm “circle” experimental point is for the lattice with *J* ~ 6.5 mT, its location is approximate.

**Fig. 5:**
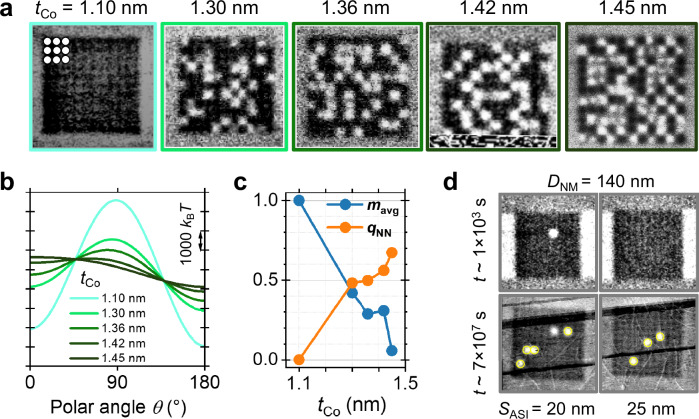
Dependence of ordering on the Co layer thickness and the nanomagnet size observed with MFM. All systems were initialized to a uniform magnetic state (black contrast) with an out-of-plane magnetic field before the measurements. **a** MFM images of lattices with *D*_NM_ = 200 nm, *S*_ASI_ = 20 nm and increasing Co thickness *t*_Co_ observed at *t* ~ 1000 s. Image frame colours correspond to those of the curves in **b**. White contrast indicates that the nanomagnets have switched. **b** Energy landscapes calculated for the lattices shown in **a**. **c** Average magnetization *m*_avg_ and local antiferromagnetic parameter *q*_NN_ as a function of *t*_Co_ determined from MFM images in **a**. **d** MFM images of lattices with *D*_NM_ = 140 nm and *t*_Co_ = 1.45 nm observed at *t* ~ 1000 s and *t* ~ 7×10^7^ s. The nanomagnets that have switched between the two times are indicated with yellow circles.

Having looked at the effect of *S*_ASI_ and time *t* on ordering in the system, we then looked at the influence of *t*_Co_ and *D*_NM_. MFM images taken at *t* ~ 1000 s of the nanomagnet arrays with *D*_NM_ = 200 nm, *S*_ASI_ = 20 nm and varying *t*_Co_ from 1.1 nm to 1.45 nm are shown in Fig. 5a. As we have seen, *t*_Co_ has a profound effect on *E*_EA_ and *E*_MC_ (Fig. 3e–g), and consequently on the ordering timescale. Indeed, as *t*_Co_ increases, the corresponding energy landscapes become more asymmetric (Fig. 5b) and, from the MFM images, we find that *m*_avg_ decreases while *q*_NN_ increases (Fig. 5c). Increasing *D*_NM_ to 200 nm provides a lower *m*_avg_ and higher *q*_NN_ (0.06 and 0.67, respectively; see data for *t*_Co_ = 1.45 nm in Fig. 5c) than for the same lattice with *D*_NM_ = 170 nm (0.47 and 0.44, respectively; see data for *S*_ASI_ = 20 nm in Fig. 4f). However, further increase of *D*_NM_ to 230 nm in the same stack results in formation of multidomain states within the nanomagnets, providing an upper limit to *D*_NM_. Conversely, a decrease of *D*_NM_ to 140 nm slows down the ordering process so that there is hardly any switching at *t* ~ 1000 s (top images in Fig. 5d) and only a few reversed magnets at *t* ~ 7 × 10^7 ^s (bottom images in Fig. 5d). We have therefore experimentally confirmed that *D*_NM_ and *t*_Co_ have a profound effect on the ordering dynamics of the lattice with *t*_Co_ requiring a precise adjustment to achieve a low enough *E*_EA_ (as shown in Fig. 3d-g). The out-of-plane anisotropy of the nanomagnets means that the state of the nanomagnets can be directly accessed with electrical readout using the anomalous Hall effect. To demonstrate this, we performed electrical measurements on lattices with *D*_NM_ = 140 and 170 nm and *S*_ASI_ = 30 and 40 nm on timescales of tens of seconds, which showed similar degrees of thermal activity to the lattices measured with MFM (Supplementary Note 6).

We have now demonstrated experimentally that the relaxation dynamics of out-of-plane spin lattices can be engineered with careful tuning of the anisotropy of and magnetostatic coupling between the nanomagnets by altering their dimensions, separation and shape, as well as the stack layer thicknesses. We have provided numerical evidence that the timescale associated with the collective dynamics can be tuned all the way from sub-seconds to years. This opens up unexplored avenues for optimising the performance of reservoir computing systems incorporating these lattices as we show in the next section.

### Tuneable-frequency reservoir computing with 2D Ising systems

The 2D Ising system is an appealing platform for a variety of computational approaches. Short-term memory and reset of the system after processing an input play a central role in brain-inspired computing^62,63^, and both of these properties can be achieved by exploiting the dynamics of *m*_avg_ and *q*_NN_ that we presented above. This ability to tune the dynamics is particularly important in reservoir and probabilistic computing where, in contrast to conventional von Neumann computing, higher frequencies are not inherently beneficial. Instead, the priority lies in matching the dynamics of the physical system to the timescale of incoming data patterns since this is the only way that the physics of the system can be effectively harnessed for data processing. To demonstrate the effect of frequency matching on the computational performance, we have simulated a signal transformation task using our 2D Ising system for reservoir computing.

Reservoir computing is a framework for neural networks that makes use of a system with non-linear behaviour called a “reservoir” to map input data into a higher-dimensional space, in which the inputs can be separated by a linear transformation. The reservoir does not need to be trained (so it is not modified itself) but does need to be a system with short-term memory and high dimensionality^62,64^, properties that are met by many physical systems^20,21,24,64–69^. Here we employ Monte Carlo simulations using the “Hotspice” package^54^ to simulate the performance of a reservoir comprising the 2D Ising system on transforming sine waves of different frequencies into a sawtooth signal, a task that is often used to test the nonlinearity of a reservoir^69,70^.

**Fig. 6:**
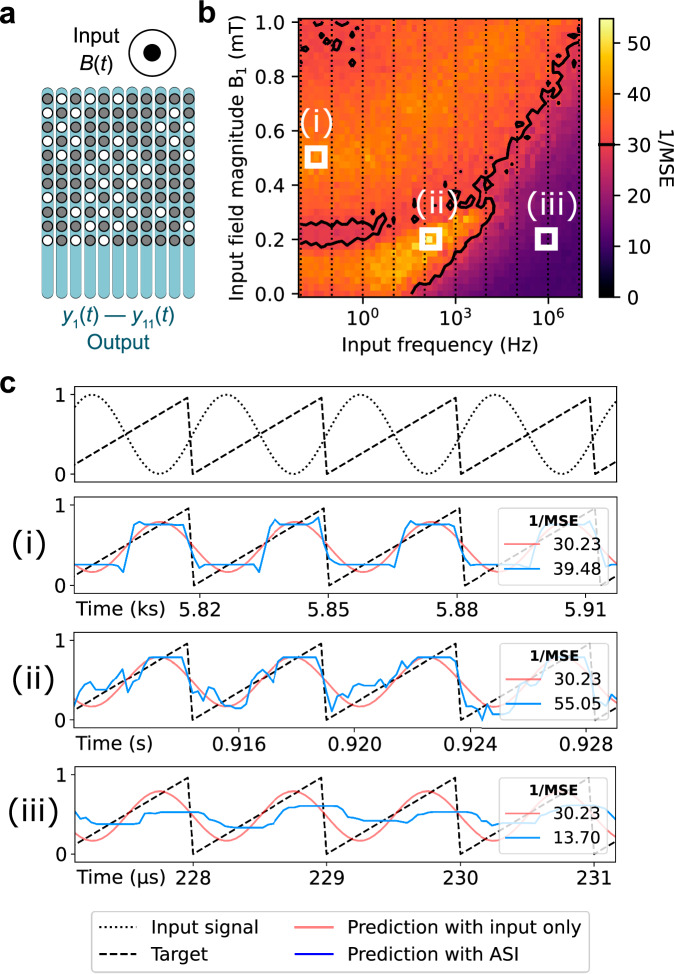
Monte Carlo simulations of the performance of an 11 × 11 lattice reservoir for a sine to sawtooth signal transformation. The parameters used for the simulations are *E*_MC_ = 2.5 *k*_B_*T*, *E*_EA_ = 20 *k*_B_*T* ± 5% and *B*_0_ = −0.35 mT. **a** Schematic of the lattice used as a reservoir. Blue readout lines indicate the row-by-row averaged magnetic state readout. **b** Inverse mean squared error 1/MSE as a function of the input magnetic field frequency and amplitude *B*_1_. Higher (lower) values in yellow (purple) indicate better (worse) performance. The black contours correspond to 1/MSE ~ 30, highlighting regions where the reservoir performs better than the linear transformation of the original input signal. **c** Temporal view of the transformation. The upper panel shows the input sinewave signal (black dotted curve) and the target sawtooth signal (black dashed line). Shown in the lower three panels are the prediction with and without the artificial spin ice (ASI) reservoir (blue and red curve, respectively) for different input frequencies with the target sawtooth signal given for comparison (black dashed line).

For the reservoir, we employed the 11×11 lattice with *E*_EA_ = 20 *k*_B_*T* ± 5% and *E*_MC_ = 2.5*k*_B_*T* (Panel 8 of Fig. 1b). We have experimentally demonstrated electrical readout of *m*_avg_ in the 11×11 lattices using the anomalous Hall effect (see Supplementary Note 6), and therefore we implemented this magnetic state readout method in the simulations. The input for the simulations was applied in the form of an out-of-plane magnetic field *B*(*t*) acting on the entire system (Fig. 6a) and was scaled such that its magnitude extends from *B*_0_ (minimum) to *B*_1_ (maximum) as shown in the top panel of Fig. 6c. Accordingly, the energy barrier term of Eq. 2 was extended to include the Zeeman term associated with the applied magnetic field as follows:

3 $$\Delta {E}_{{\mbox{j}}}\left(t\right)={E}_{{{\rm{EA}}}}\left(1+{{\rm{rand}}}\left(j\right)\right)+{E}_{{{\rm{MC}}}}{\sum}_{i,j}{S}_{i}{\left(t\right){D}_{{ij}}S}_{j}(t)+{E}_{{{\rm{Zeeman}}}}\left(B\left(t\right),j\right)$$

The average magnetization of each lattice column was used for the readout *y*_i_(*t*) (Fig. 6a). A discussion about the experimental feasibility of such a grid of local readouts is given in Supplementary Note 7. The readout was used to perform a linear regression $o\left(t\right)={\sum }_{i=1}^{n}{w}_{i}{y}_{i}\left(t\right)$ and transformation^71,72^, where *i* is the nanomagnet array column number, *w*_i_ is the associated weight, $o\left(t\right)$ is the predicted signal and *n* = 11 is the array dimension. The inverse mean squared error (1/MSE) between $o\left(t\right)$ and the desired result (the sawtooth) was used as a performance metric.

The 1/MSE for a range of frequencies and amplitudes of the input magnetic fields *B*_1_ is shown in Fig. 6b. The black contour indicates a threshold of 1/MSE ~ 30, which can be achieved using only a linear transformation without a reservoir. The best results (ii) are achieved at the input frequency of ∼100 Hz. For other frequencies (e.g. at (i) 0.05 Hz and (iii) 100 kHz), the performance is noticeably worse and cannot be improved to the same level by altering the input field magnitude *B*_1_ (Fig. 6b). Interestingly, the best performance is achieved at frequencies around the transition between the first (*m*_avg_ > 0) and the second (*m*_avg_ ~ 0, *q*_NN_→1) ordering stages of the lattice (Panel 8 of Fig. 1b). A low-frequency input corresponds to the longer relaxation time (green-shaded region of Panel 8 in Fig. 1b) and allows the system to reach the same state with *m*_avg_ ~ 0 regardless of the input data. This results in a square rather than a sawtooth signal after the transformation (blue trace in panel (i) of Fig. 6c). For a high-frequency input (panel (iii) in Fig. 6c), the lattice cannot respond fast enough to the changes in the signal and cannot reproduce the rise or the sudden drop of the sawtooth. At an optimal input frequency (ii), the system achieves an MSE comparable to other magnetic reservoirs^69^. The performance can be further improved by increasing the size of the lattice or introducing a gradient in the effective anisotropy (see Supplementary Note 8).

In summary, we have demonstrated that the performance of the 2D Ising reservoir depends on how well the ordering dynamics matches the frequency of the input. This is characteristic for this type of computation and, therefore, the ease with which one can engineer the relaxation timescale in the 2D Ising lattices is highly advantageous. This avoids the otherwise necessary and computationally expensive preprocessing of the input data^65,68,70,73^.

## Discussion

We have shown with Monte Carlo simulations that the timescale of the relaxation dynamics in 2D Ising lattices can be tuned by varying the effective anisotropy and magnetostatic coupling, and that this can be exploited to achieve an enhanced performance of these lattices for reservoir computing. This information allowed us to fabricate experimental arrays of magnetostatically-coupled perpendicular nanomagnets with the desired properties by engineering the energy landscape through careful selection of materials and geometries.

We have shown that we can achieve a high degree of spontaneous ordering in arrays of out-of-plane nanomagnets. Nevertheless, achieving such a high level of ordering on an even shorter timescale as well as reaching *q*_NN_ = 1 would be beneficial. Placing our experimental results on the ordering phase diagrams in Fig. 2 reveals a way to do so. Indeed, Region IV with *q*_NN_ ~ 1 is located below the experimental points (circle, triangle and cross in Fig. 2) indicating sufficient *E*_MC_ and excessive *E*_EA_. Therefore, in order to achieve a perfect ordering at a given timescale, we need a method to tune *E*_EA_ with more precision. While we have shown that *E*_EA_ can be reduced by increasing the magnetic layer thickness *t*_Co_, going beyond the nominal sub-0.1 nm precision of *t*_Co_ used in this work is challenging. Therefore, to control the perpendicular magnetic anisotropy in Co/Pt multilayers more precisely in the future, one can use thermal annealing^74–77^. Furthermore, laser annealing^78,79^ would provide a means to locally modify *E*_EA_ and create spatially complex designs.

In addition to making permanent modifications to the magnetic properties, *E*_EA_ can be temporarily changed by applying current. The generated spin-orbit torque does not lift the degeneracy of “up” and “down” magnetic states in the out-of-plane easy axis geometry^80,81^. Therefore, on application of a current, the Joule heating and spin-orbit torque (requiring the lattice to be placed on an appropriate layer) lower the energy barrier to switching without interfering with the *E*_MC_-driven ordering. Experimental confirmation of this is detailed in Supplementary Note 9. Using voltage and current to reduce *E*_EA_ would provide a way to dynamically change the relaxation timescale of the lattice, opening the way to create systems with an adaptive temporal response, which is a cornerstone of information processing in the brain^82,83^ and brain-like computing^84,85^. In addition, by engineering the current density distribution (as discussed in Supplementary Note 7) or by applying a voltage to individual nanomagnets^86^, a more local electrical control could be achieved.

We have shown that, by careful choice of the layer number and thicknesses in out-of-plane artificial spin lattice, a magnetostatic coupling energy *E*_MC_ of tens of *k*_B_*T* can be obtained. The ability to create systems with such high coupling provides an alternative, more energy efficient method to link the nanomagnets compared with the external electronic connections often used in probabilistic computing schemes^49,87^ or for time multiplexing in reservoir computing^65,68^. For such applications, a further increase in *E*_MC_ could be beneficial and can be accomplished by adding a soft magnetic layer with in-plane anisotropy underneath the out-of-plane lattice^28^. We tested this approach on square arrays of nanomagnets with *D*_NM_ = 140 nm and observed a substantial increase in the spontaneous ordering (Supplementary Note 10). Another approach to increase *E*_MC_ is to optimise the shape of the nanomagnets (Supplementary Note 11). Such control of the coupling by altering the shape is uniquely suited to out-of-plane nanomagnets since, for in-plane magnets, a change of shape tends to give a change in the distribution of the demagnetization field.

This freedom to control both *E*_EA_ and *E*_MC_ locally will provide a means to create lattices with novel emergent properties. In terms of fundamental science, this means that the thermodynamics can now be studied with different lattice geometries to reveal new phases and phase transitions. In addition to creating thermally-active out-of-plane systems, it will now be possible to pattern mixed lattices incorporating both thermally-active in-plane and out-of-plane nanomagnets. These do not necessarily have an equivalent in bulk crystal systems and are likely to display unusual collective phenomena.

In terms of applications, these time-dependent artificial spin lattices of perpendicular nanomagnets offer an exciting platform for reservoir or probabilistic computing in square or other more complex geometries. The fact that these systems can be electrically interfaced, adapted to the input frequency and have short-term memory based on thermal activity, means that they have all the properties required for next generation computing.

Our work therefore opens vistas across the fields of spintronics, reservoir computing and artificial spin ice, providing precise control of the cooperative behaviour and a flexibility in the design that can be finely tuned for different computing applications.

## Methods

### Sample fabrication

The films were deposited at room temperature onto high-resistivity Si wafers with a natural oxidation layer using DC magnetron sputtering. A base pressure of ≤1 × 10^−8^ Torr and Ar gas pressure of 3 mTorr were used for the sputtering. The deposited films were processed into devices with electron beam lithography and Ar ion milling. 50 nm-thick 2% hydrogen silsesquioxane was used as an electron beam resist to achieve high-resolution patterning of the nanomagnets. A milling current of 60 mA and angle of 5° were used. Poly(methyl methacrylate) 4% 950k was used as an electron beam resist for patterning the Hall bars and electrodes.

### MFM measurements

All MFM measurements were performed at room temperature. Low-moment Bruker MESP-V2-LM probes were used. The samples were initialized by applying three 5-second-long pulses of a 0.9 T out-of-plane magnetic field, sufficient to saturate them and give a uniformly magnetized state on removing the magnetic field before the MFM measurements were carried out. Care was taken to make sure that there was no lateral magnetic field component or remanent magnetization associated with the magnetic field source that would affect the state of the nanomagnet arrays. All MFM scans were performed at zero field.

### Energy landscape calculations

The energy landscapes were calculated in Mathematica using a custom code. The profiles were calculated for the polar angle of magnetic moment of the nanomagnet going from 0° to 180° in 1° steps. To obtain the saturation magnetization of 1063 kA/m and interfacial anisotropy of 1.46 mJ m^−2^ used for the energy landscape calculations, the thin films were measured using superconducting quantum interference device vibrating sample magnetometry (SQUID VSM). Details of the energy landscape calculations are given in Supplementary Note 4.

### “Hotspice” Monte Carlo simulations

The relaxation of the 2D Ising lattices and reservoir capability were simulated in Python using a custom “Hotspice” Monte Carlo code and employing a magnetic moment of 2.37 × 10^−16 ^A m^2^ for each spin and a temperature of 300 K. 600 input periods were used for the linear regression. 400 input periods were used to test the performance of the signal transformation. Details of the simulations are given in Supplementary Note 1.

## Supplementary information

Peer review file

Supplementary Material

## Data Availability

The data that support the findings of this study are openly available in Zenodo at 10.5281/zenodo.15882564.

## Code availability

Access the Hotspice open-source code at https://github.com/bvwaeyen/Hotspice. The codes used to generate data in this manuscript can be accessed at 10.5281/zenodo.15882564.
