# Supplementary material for: Perpendicular-anisotropy artificial spin ice with spontaneous ordering: a platform for reservoir computing with flexible timescales
Source: Commun Eng. 2025 Nov 3;4:183. doi: 10.1038/s44172-025-00499-y (PMC12583449; doi:10.1038/s44172-025-00499-y)
Supplement: Supplementary file 2 — Supplementary Material [file 44172_2025_499_MOESM2_ESM.pdf]

SUPPLEMENTARY INFORMATION

Perpendicular-anisotropy artificial spin ice with spontaneous ordering: a platform for reservoir computing with flexible timescales

Aleksandr Kurenkov[1,2], Jonathan Maes[3], Aleksandra Pac[1,2], Gavin Martin Macauley[1,2], Bartel Van Waeyenberge[3], Aleš Hrabec[1,2] and Laura Jane Heyderman[1,2]

[1]Laboratory for Mesoscopic Systems, Department of Materials, ETH Zurich, 8093, Zurich, Switzerland
[2] PSI Center for Neutron and Muon Sciences, 5232 Villigen PSI, Switzerland
[3]DyNaMat, Department of Solid State Sciences, Ghent University, Belgium

Supplementary Note 1: "Hotspice" Monte-Carlo simulation details

The simulations were performed using the Hotspice[1] software package, which models the system as a lattice of dipolar-coupled Ising spins at a temperature T. The spins switch randomly as determined by the Néel-Arrhenius law $\tau_{\mathrm{j}}(\mathrm{t}) = \tau_0 \exp\left(\frac{\Delta E_j(t)}{k_B T}\right)$. The energy barrier to switching of an isolated spin $\Delta E = E_{\mathrm{EA}}$. However, the interaction with other nanomagnets (E_{MC}) or an external magnetic field (E_{Zeeman}) affects the energy landscape and thus the effective barrier. In the limit of a zero-energy barrier, $\Delta E \to 0$, the switching time $\tau_j(t) \to \tau_0$. We choose $\tau_0 = 10^{-10}$ s, which is the timescale of the natural period of gyromagnetic precession of the magnetization about the easy axis of the nanomagnet[2].

The time evolution of the magnetic state of the lattice was evaluated stepwise, one spin switch at a time. Which spin switches next and how much later after the previous switching event it happens, is determined as follows. First, $\Delta E(t)$ is calculated for each nanomagnet individually, based on the lattice magnetization state. The corresponding average switching times $\tau_{\mathrm{j}}(t)$ readily follow from the Néel-Arrhenius law. Then, for each magnet j, a random time Δt_j is taken from an exponential distribution with mean value τ_j. The nanomagnet with the smallest switching time $\Delta t = \min_j(\Delta t_j)$ will then switch if $\Delta t < t_{\mathrm{max}}$, and the elapsed time t increases by $\min(\Delta t, t_{\mathrm{max}})$. The purpose of t_{max} (default value is 1 second) is to avoid loss of accuracy for time-dependent external fields. For example, when a sinusoidal signal of frequency f is applied to the lattice $t_{\mathrm{max}} = 20/f$ ensures the simulation captures the waveform in enough detail.

Supplementary Note 2: The mechanism of the ordering slowdown after m_{avg} ~ 0 is reached

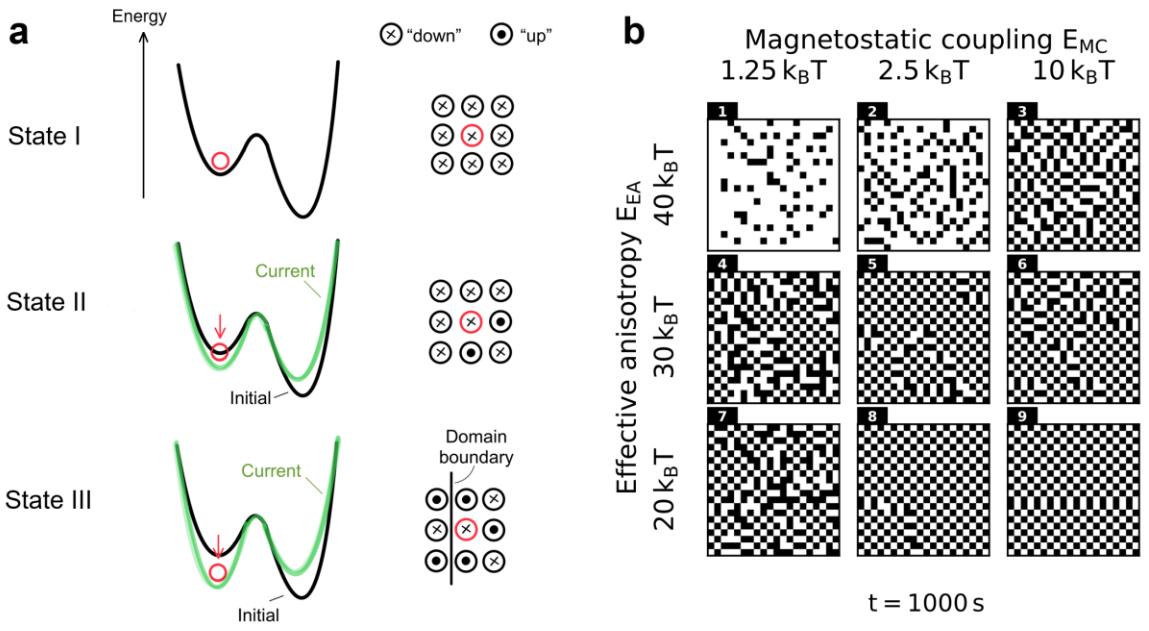

Figure S1. (**a**) Schematics of the energy landscape of the nanomagnet highlighted in red depending on the magnetic state of its neighbours. (**b**) Magnetic states of a 20×20 lattice at *t* = 1000 s. White (black) contrast corresponds to the nanomagnets with "up" ("down") magnetic states.

The schematics in Figure S1a outline the energy-driven mechanism of the ordering slowdown after domain boundaries in the system have formed.

State I: the uniformly initialized state at *t* = 0. It is easy for the central red spin to switch, as well as for its neighbours. These initial switching events start happening all over the system, eventually forming magnetically ordered areas (domains).

State II: assuming two of the neighbours of the red spin have switched, the red spin sinks deeper into its energy minimum. The stronger E_{MC} is, the more pronounced this change in the energy minimum will be. It is now harder for the red nanomagnet to change its magnetic state.

State III: after some additional switching in the lattice, a domain boundary has formed, and the red spin is now a part of it. In order to reach perfect ordering, the red spin (or other similar spins in the domain boundary) would have to switch. However, it is now much more energetically expensive to switch than during the initial phase of individual switching events since the energy well is now deeper. This does not prevent the ordering process but significantly slows it down, resulting in the change of slope of q_{NN} (the orange traces) in Figure 1 of the main text.

Figure S1b is the same as Figure 1c of the main text but for a larger 20×20 lattice. The extended lattice provides an additional overview of the formation and expansion of domains. This process is reminiscent of domain wall coarsening—a process where magnetic domains within a material grow over time, leading to a reduction in the total number of domain walls. Here, however, we include superparamagnetic activation energies that are typically neglected in the models of domain wall coarsening.

The slope of the orange line gets steeper in the range of t from ~10^1 to ~10^4 s in Panel 9 of Figure 1b of the main text. Domain wall dynamics can also explain this feature. At $t \sim 10^0$ s, the domain walls have already formed in the system and any further increase in the local antiferromagnetic parameter occurs through the reduction of the total length of these domain walls. This reduction happens via a random walk mechanism as domain walls exit the system. Here, the distance that a domain wall travels is proportional to $(t/T)^{\alpha}$, where T is the switching frequency of an individual domain wall magnet, t is the elapsed time, and α represents the power coefficient. On a logarithmic time scale (x-axis), this dependence appears as a sudden increase in q_{NN} . In Panel 9, the switching frequency T of domain wall magnets is on the order of several seconds, which leads to the step near 1000 seconds. At lower E_{MC} values, this plateau becomes less distinct because reduced coupling makes domain walls less stable, blurring the transition.

The spread in q_{NN} and m_{avg} at higher times, especially noticeable in Panels 4 and 7 of Figure 1b of the main text, is also a consequence of the logarithmic scale on the x-axis. As the system approaches equilibrium, thermal fluctuations lead to switching of the nanomagnets occurring at a fixed frequency. On a logarithmic time axis, this switching becomes increasingly compressed into the same x-interval as time progresses, which appears as the increased vertical spread in q_{NN} and m_{avg}.

Supplementary Note 3: Dependence of m_{avg} and q_{NN} on E_{MC}, E_{EA} and time following uniform initialization

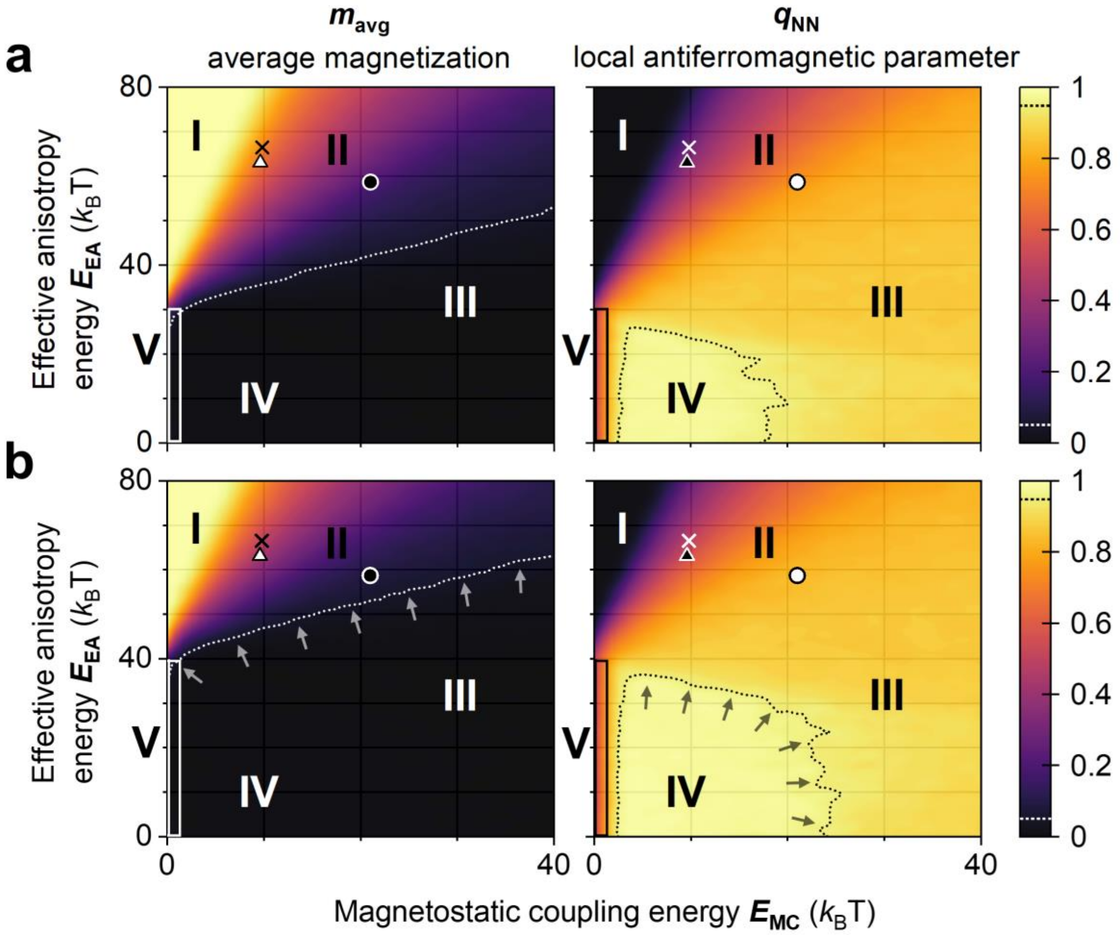

Figure S2. Phase diagrams of the average magnetization m_{avg} and local antiferromagnetic parameter q_{NN} as a function of the effective anisotropy E_{EA} and magnetostatic coupling E_{MC} (**a**) at $t \sim 1000$ s and (**b**) at $t \sim 7\times10^7$, which is ~27 months. The dotted lines are at values of 0.05 (white) and 0.95 (black). The changes in the position of the lines are highlighted by arrows. The labels for the five regions (I – V) and the experimental points (triangle, cross, circle) are the same as in Figure 2 of the main text. The vertical box showing Region V is the same in all panels.

Phase diagrams of the average magnetization m_{avg} and local antiferromagnetic parameter q_{NN} at $t \sim 1000$ s (Figure S2a) and $t \sim 7\times10^7$ (Figure S2b). Note the shifting of the borders of Regions III, IV and V as time increases. The border of Region V in Figure S2b is now located at $E_{EA} \sim 41$ k_BT, because nanomagnets with this effective anisotropy have an average switching time of ~ 7×10^7 s. Note that these phase diagrams were calculated assuming that there is no exchange coupling between the nanomagnets, so $J = 0$. Therefore, since the $S_{ASI} = 20$ nm 'circle' experimental point is for the lattice with $J \sim 6.5$ mT, its location is approximate.

Supplementary Note 4: Calculation of energy landscape of coupled Co/Pt nanomagnets

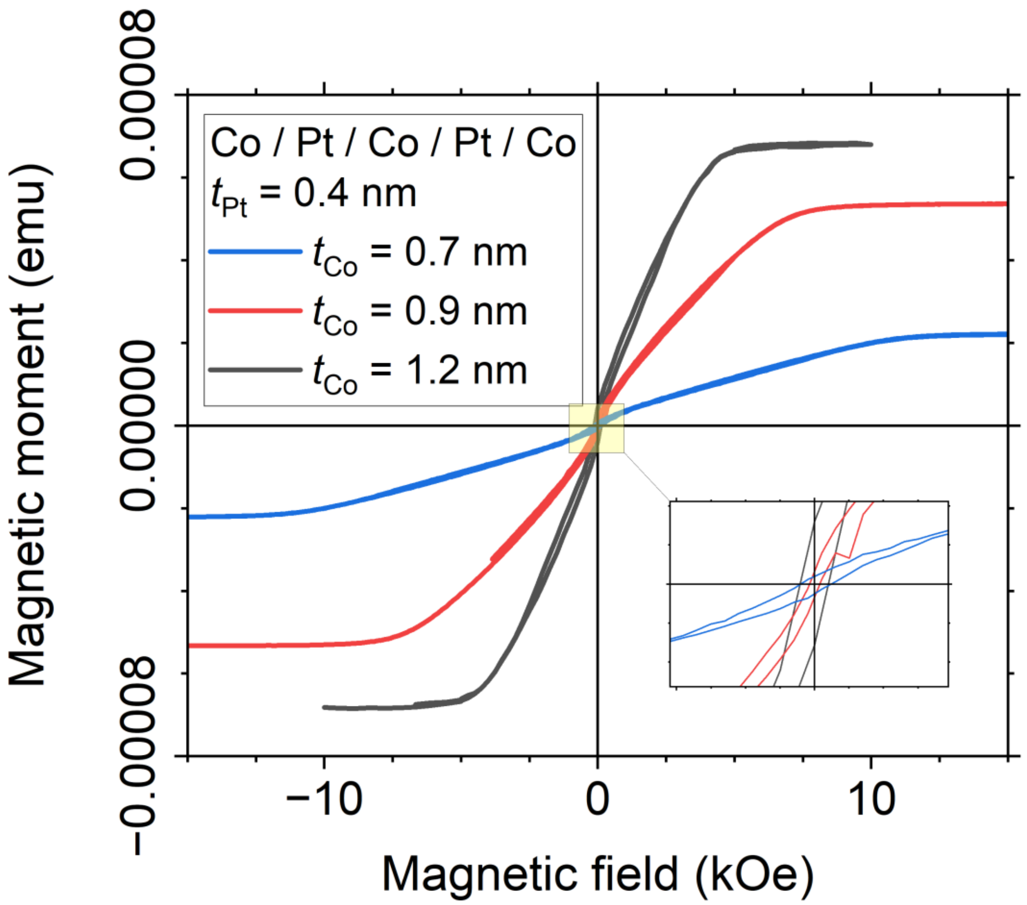

Figure S3. In-plane superconducting quantum interference device - vibrating sample magnetometry (SQUID-VSM) measurements of thin films with different Co thicknesses.

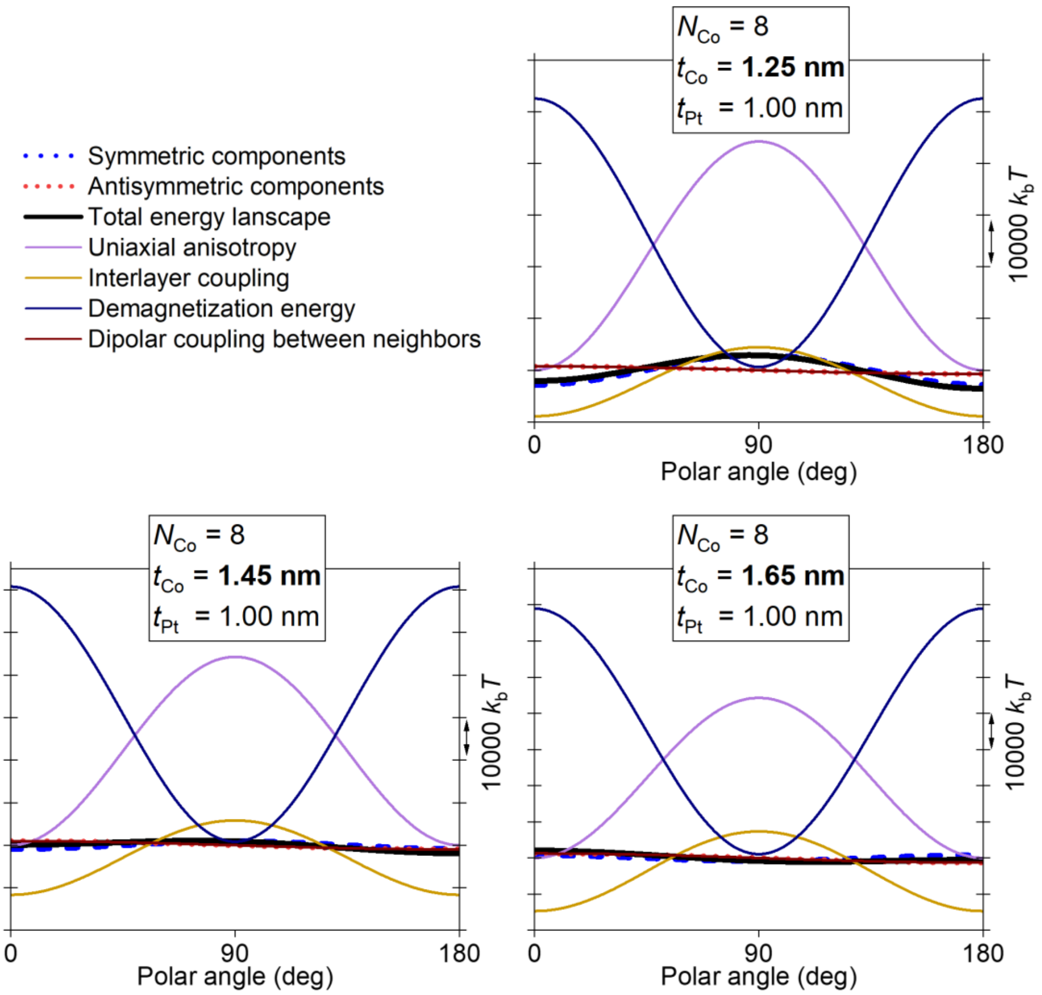

Figure S4. Energy landscapes for the same cases shown in Figure 3(e)-(g), but now with all four energy terms.

The energy landscapes were calculated in Mathematica for polar angles from 0° to 180° in 1° steps. Four energy terms were considered associated with (i) the uniaxial anisotropy, (ii) the demagnetization, (iii) the interlayer magnetostatic interaction within the nanomagnet and (iv) the dipolar coupling to the neighbouring nanomagnet. The first three terms contribute to effective anisotropy E_{EA} and the last one is the only contributor to the magnetostatic coupling term E_{MC}. A uniaxial anisotropy of 1.46 mJ/m^2 was determined from vibrating sample magnetometry measurements of the multilayer films with N_{Co} = 3, t_{Co} = 1.2 nm and t_{Pt} = 0.4 nm (Figure S3). For the other three terms, a saturation magnetization of 1063 kA/m, derived from the same measurements, was included. In-plane hysteresis loops measured with superconducting quantum interference device - vibrating sample magnetometry (SQUID-VSM) for stacks with t_{Co} = 0.7, 0.9 and 1.2 nm are shown in Figure S3. Here, a decrease in the out-of-plane anisotropy field with increasing t_{Co} is observed, as seen by the change in slope in the hysteresis loops.

The dipolar coupling between each pair of nanomagnets was calculated by direct integration. The interaction between the layers within one nanomagnet was calculated following Dmytriiev *et al*. (2010)[3]. The demagnetizing energy was calculated using the demagnetization tensor from Joseph (1966)[4].

Shown in Figure S4 are the energy landscapes of Figure 3(e)-(g) with all four energy components plotted along with E_{EA}, E_{MC} and (E_{EA}+E_{MC}). From the relative magnitudes of the four energy

components compared to the final landscape, one can appreciate the precision required in balancing the demagnetizing energy with effective anisotropy to achieve a desired landscape. Another interesting observation is the non-negligible role of the interlayer coupling within a nanomagnet (yellow trace).

Note that the energies obtained in these energy landscape calculations are not the ones used in the “Hotspice” Monte-Carlo simulations. This is because the energy landscape simulations overestimate the energy as they assume a simplified reversal model of coherent rotation of the magnetization, rather than domain wall nucleation and propagation. Nevertheless, this simple model gives important information of the dependence of the energy landscape on the lattice parameters. For the Monte Carlo simulations, we cover a parameter space that is relevant to our experimental systems.

Supplementary Note 5: Fitting of the experimental MFM data for D_{NM} = 170 nm with Monte Carlo simulations

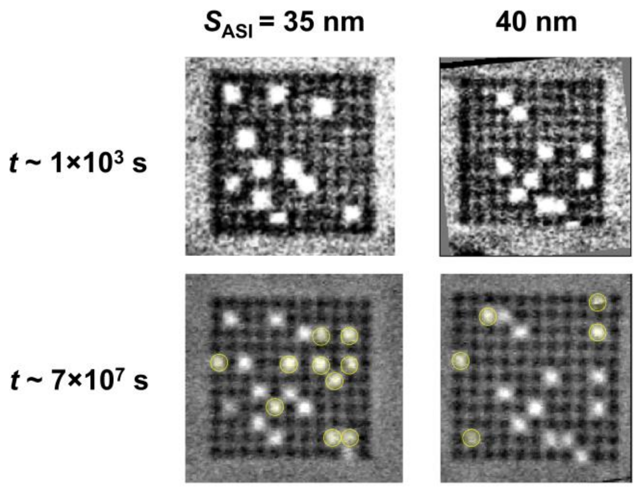

Figure S5. MFM images of lattices with D_{NM} = 170 nm, t_{Co} = 1.45 and S_{ASI} = 35 and 40 nm at t = 1000 s and t = 7×10^7 s. The magnets that have switched between these observations are highlighted with yellow circles.

In Figure S5, MFM images are given for the lattices with D_{NM} = 170 nm and nanomagnet separations, S_{ASI}, of 35 and 40 nm. The measurements were performed in the same way and at the same time as the images in Figure 4 of the main text. This data was used to determine the corresponding m_{avg} and q_{NN} in Figure 4f, as well as q_{2NN} and q_{3NN}.

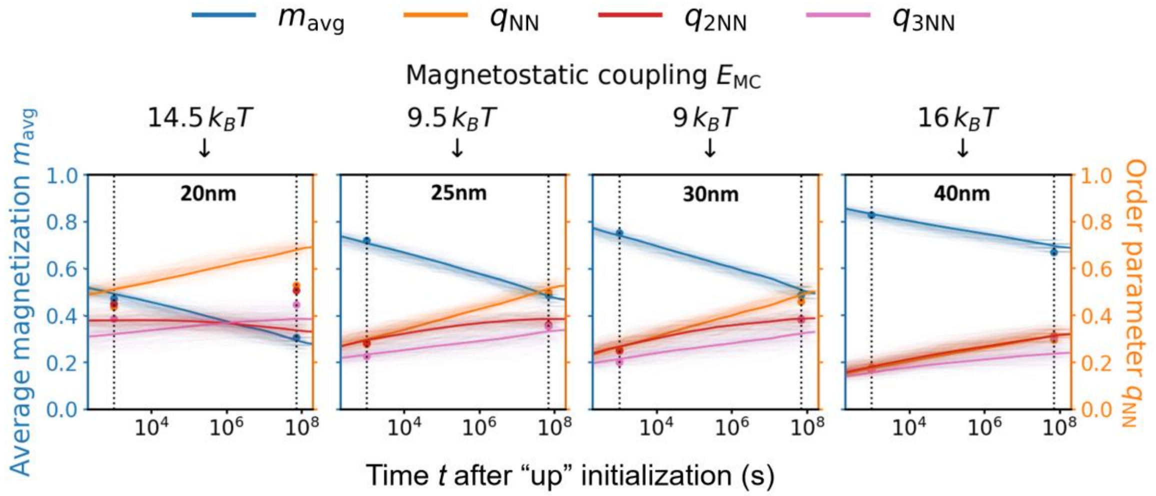

Figure S6. Evolution with time of the average magnetization m_{avg} and local antiferromagnetic parameters q_{NN}, q_{2NN}, q_{3NN}. The fine lines are calculated using Monte Carlo simulations assuming point dipoles and no exchange coupling. The bold lines are their averages. The points are experimental data. E_{MC} obtained from the fitting are shown in the figure. E_{EA} obtained from the fitting is ~65.0 k_BT.

We then performed a fitting of the Monte Carlo simulated curves to the experimentally determined values of m_{avg}, q_{NN}, q_{2NN} and q_{3NN} at t ~ 1000 s and t ~ 7×10^7 s. The simulation was carried out in exactly the same way as to calculate relaxation dynamics graphs shown in Figure 1b of the main text, and we used E_{EA} and E_{MC} as fitting variables. This fitting was performed using a weighted least-squares method, where the weights for the variables m_{avg}, q_{NN}, q_{2NN} and q_{3NN} at each time point t were determined based on the variability observed in the Monte Carlo simulations. Specifically, each variable was assigned a weight inversely proportional to its standard deviation at that time. The smaller the standard deviation, the higher the weight, implying a higher fit quality. The results are shown in Figure S6.

As can be seen in Figure S6, a better fit for S_{ASI} = 25 and 30 nm could be achieved than for S_{ASI} = 20 nm. As explained in the main text, this could be due to a residual Co layer between the nanomagnets that results in some ferromagnetic exchange coupling between them. To account for this, we introduced exchange coupling J into the fitting procedure. This J gives a reduction in the nearest-neighbour dipolar coupling. We also accounted for the finite size of the nanomagnets by adding a 1/r^5 term to D_{ij} (Eq. 2 of the main text) as described in Reference [5]. This resulted in a significantly better fit for the S_{ASI} = 20 nm case, as shown in Figure S7a. As expected, the fitting procedure returned a non-zero exchange coupling of ~ 6.5 k_BT for the lattice with S_{ASI} = 20 nm and near-zero exchange coupling for lattices with S_{ASI} = 25 and 30 nm.

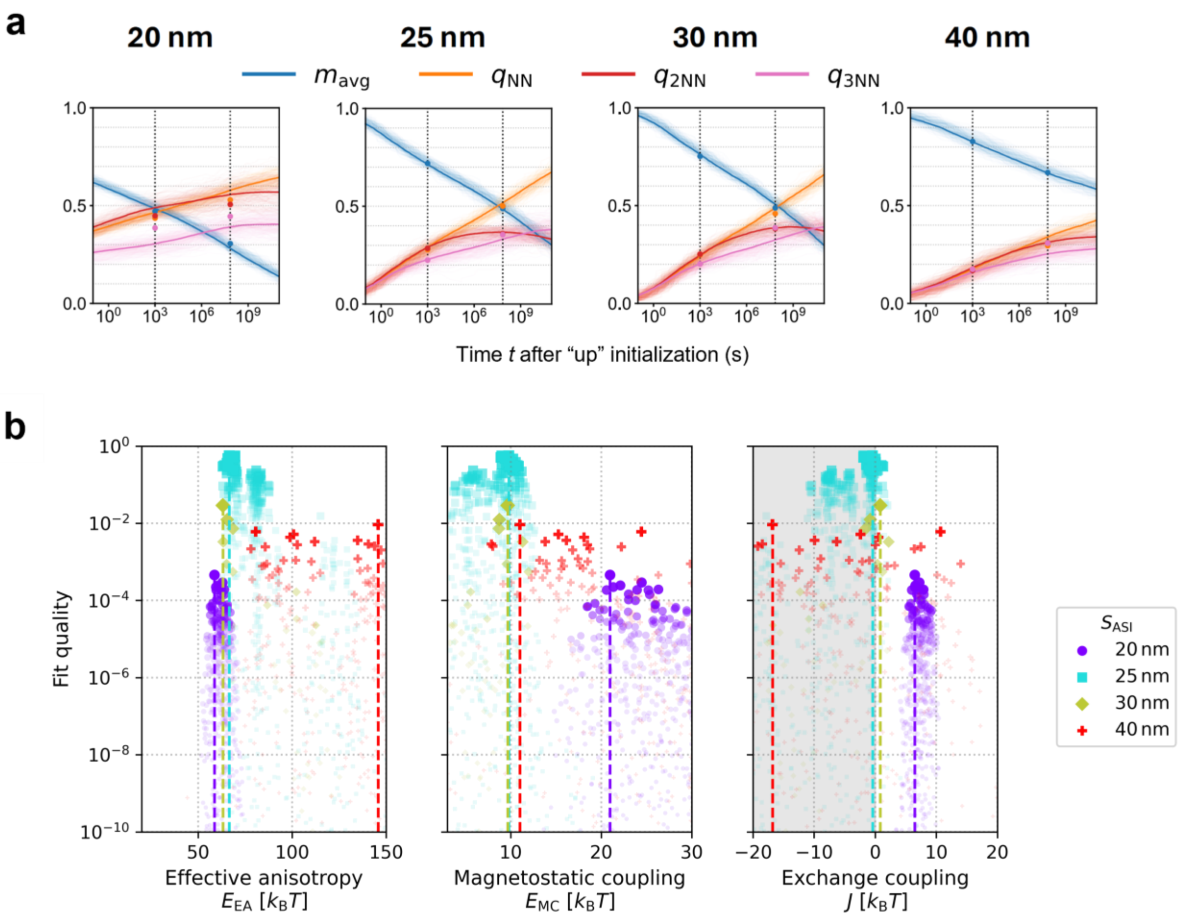

Figure S7. Fitting for the experimental data that accounts for non-zero exchange coupling J. Panel (**a**) shows the fitted results; the lines and points are as in Figure S6. Panel (**b**) shows how well the Monte Carlo simulation fits the experimental data at a given E_{EA}, E_{MC} and J value (left, middle and right panel, respectively). A larger y-axis value (fit quality) indicates a better fit. For example, for

S_{ASI} = 20 nm (purple points) and E_{EA} (left plot), the highest y-values are concentrated around E_{EA} = 60 k_BT, indicating that the best fit for this lattice can be achieved at E_{EA} of ~60 k_BT. Greyed-out area represents antiferromagnetic coupling (negative *J*), which is considered non-physical in our system.

Despite the introduction of *J*, we could not obtain a good fit for the S_{ASI} = 40 nm. Figure S7b shows how well the experimental data can fitted at a given E_{EA}, E_{MC} and *J* (left, middle and right panel, respectively). In particular, each data point in Figure S7b corresponds to a fit of the experimental data to the Monte Carlo simulation (fit quality), and the larger the y-axis value, the better the fit. Whereas for S_{ASI} = 20, 25 and 30 nm, the best fits are mainly concentrated in a rather narrow range of E_{EA}, E_{MC} and *J* (cyan, purple and green points), this is not the case for S_{ASI} = 40 nm (red points). Indeed, for S_{ASI} = 40 nm, fits of equal quality could be obtained for several E_{EA}, E_{MC} and *J*, with many of the red points taking similar y-axis values.

In Figure S8a, additional evolution of magnetic states of the array with D_{NM} = 170 nm and S_{ASI} = 25 nm during a 5-hour continuous scanning is shown. 13 nanomagnets switch before *t* = 4 min and 3 additional nanomagnets switch between *t* = 4 min and *t* = 312 min. This happens because of the ordering slowdown, as shown in Figure S7a. Here, the change in m_{avg} and q_{NN} per time interval gradually decreases with time, indicating a decrease in the number of newly switched nanomagnets.

During the original 2-year experiment (Figure 4e), the sample was kept in a field-free environment at all times except for the two instances when it was measured, whereas the MFM tip was continually scanning across the sample during the new 5-hour experiment displayed in Figure S8a. Therefore, the simulated 5-hour traces in the figure below account for the presence of the MFM tip in the form of a nonzero external field. Since both the tip and sample are initially magnetized in the same direction, the stabilizing influence of the MFM tip results in a slight shift to longer timescales. An average 50 μT field was found to correspond well with the observed 5-hour data, which is shown in Figure S8b. As before, the new experimental data points fall within one standard deviation of the simulated traces.

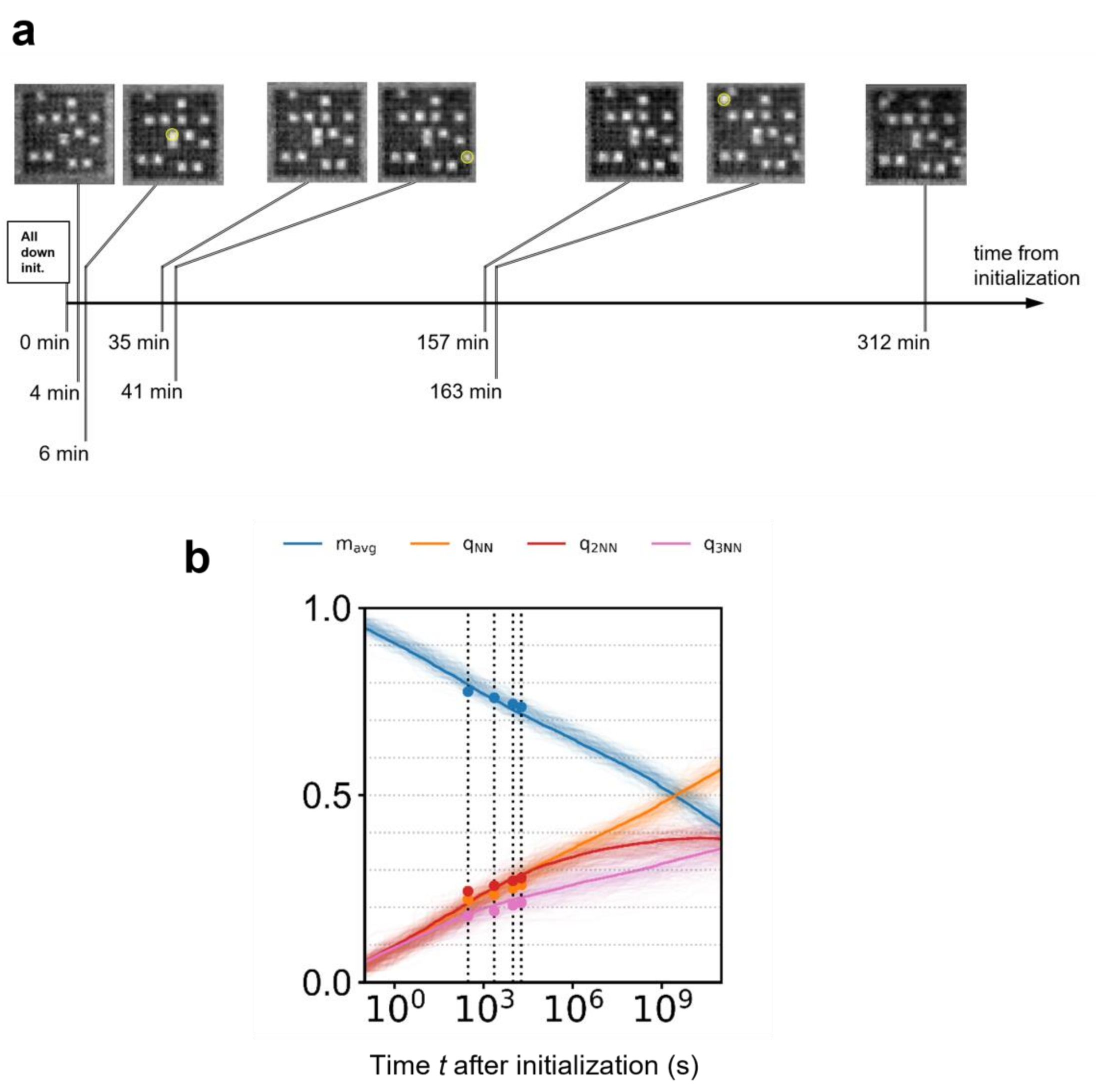

Figure S8. (a) MFM images at additional times for an array with D_{NM} = 170 nm and S_{ASI} = 25 nm. Yellow circles highlight magnets that have switched between the corresponding and the previous measurement. (b) Simulations of the 5-hour measurement. The 4 data points correspond to the 3 observed switches and the final MFM image of Figure S8a. Simulated traces use the same effective anisotropy EEA and magnetostatic coupling EMC as the 2-year measurement in Fig. 4e, but now includes an external 50 μT field that models the average influence of the MFM tip.

Supplementary Note 6: Experimental electrical readout of out-of-plane artificial spin lattice

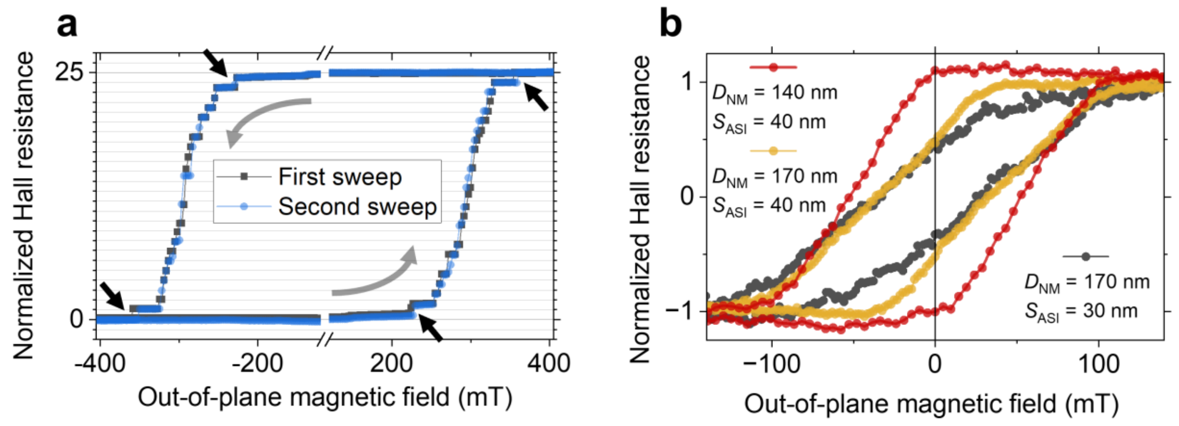

Figure S9 | Electrical readout of square lattices of perpendicular nanomagnets. The lattices were placed on Hall bars. The magnetic field was swept while probing anomalous Hall voltage. The grey arrows indicate the direction of the magnetic field sweep. (**a**) Hall resistance measurements on varying the out-of-plane magnetic field for a lattice of 5×5 magnets with t_{Co} = 1.2 nm and D_{NM} = 800 nm that did not show spontaneous switching at the timescale of the experiment. The readout was normalized from 0 to 25 to highlight the 25 binary switching events associated with the 25 individual nanomagnets. The black arrows point to the first and last switching event when increasing and decreasing the magnetic field. (**b**) Hall resistance measurements on varying the out-of-plane magnetic field for a lattice of 11×11 magnets with different nanomagnet diameters D_{NM} and separations S_{ASI}. A Keithley 6221 current source and 2182 nanovoltmeter were used for electrical measurements.

Electrical interfacing is essential for practical applications as well as for fundamental investigations of the magnetization dynamics in artificial spin ice[6], providing a means to probe the dynamics in systems of any size in a time-resolved fashion[7]. However, electrical interfacing is rarely used to probe nanomagnets with in-plane easy axis since this cannot be carried out without the help of additional layers[6], second-harmonic readout[8], external probing of the magnetic field[8] or performing measurements at temperatures below 50 K[9].

In contrast to in-plane systems, the out-of-plane anisotropy of the nanomagnets in our artificial spin lattice allows us to directly perform electrical readout as they are. To demonstrate this, we placed several different lattices on Hall bar electrodes and applied a small probing current of ~4×10^{9} A/m^2. The generated transverse anomalous Hall voltage was then divided by the current to obtain the Hall resistance R_{Hall}, which represents the magnetic state of the system and is approximately equivalent to m_{avg} (the difference between R_{Hall} and m_{avg} is discussed in Supplementary Note 7).

We first measured a 5×5 square lattice of circular nanomagnets with D_{NM} = 800 nm patterned from a (Co [1.2] / Pt [0.2])$_2$ / Co [1.2] multilayer, which did not show spontaneous ordering in the MFM images. We swept the out-of-plane magnetic field while probing R_{Hall} (Figure S9a). The readout was normalized from 0 to 25 in order to highlight the 25 binary switching events associated with each of the 25 nanomagnets. The first (last) switching event in each of the branches happens noticeably earlier (later) than the rest (shown by black arrows in Figure S9a). This may be an indication of the magnetostatic coupling between the nanomagnets as the first (last) switching

event is the most (least) energetically favourable due to the magnetic configuration of the neighbouring nanomagnets. However, this system is not thermally active at the measurement timescale of tens of seconds. The absence of thermal activity is apparent from the same value of R_{Hall} (and thus m_{avg}) for the remanent and saturated state so that, once the field is removed, the lattice remains frozen.

We then performed the same measurement on 11×11 square lattices with D_{NM} = 140 and 170 nm, S_{ASI} = 30 and 40 nm, and a multilayer stack of (Co [1.45] / Pt [0.8])$_6$ / Co [1.45] (Figure S9b). We know that these lattices show spontaneous switching at the timescale of seconds (see "Control of relaxation timescales in experimental dipolar-coupled 2D Ising systems" section of the main text). We also know that these nanomagnets are single-domain and have a perpendicular easy axis from the MFM data (Figure 4a and Figure 5d of the main text). A remanent R_{Hall} (~m_{avg}) that is lower than saturation is thus only possible due to spontaneous antiparallel magnetic ordering of the nanomagnets, and a smaller remanence indicates that more nanomagnets have switched. For the sample with D_{NM} = 140 nm and S_{ASI} = 40 nm (red loop in Figure S9b), the lattice remains in the saturated state after the field is removed, whereas increase of D_{NM} to 170 nm results in a smaller remanent R_{Hall} (m_{avg}) and onset of spontaneous switching (yellow loop in Figure S9b). Decreasing S_{ASI} from 40 nm to 30 nm for D_{NM} = 170 nm further lowers the remanent R_{Hall} (~m_{avg}) (dark grey curve in Figure S9b).

Therefore, as we go from lattices with weaker magnetostatic coupling between the nanomagnets to those with stronger coupling (D_{NM}/S_{ASI} = 140 /40 nm → D_{NM}/S_{ASI} = 170/40 nm → D_{NM}/S_{ASI} = 170/30 nm), we observe a decrease in both m_{avg} (measured by MFM) and remanent R_{Hall} (measured electrically). The trends observed in the electrical measurements are thus consistent with the MFM results, providing a justification for using electrical reservoir readout in the simulations of a 2D Ising reservoir in the "Tuneable-frequency reservoir computing with 2D Ising systems" section of the main text.

Supplementary Note 7: Simulation of local electrical readout with multiple electrodes

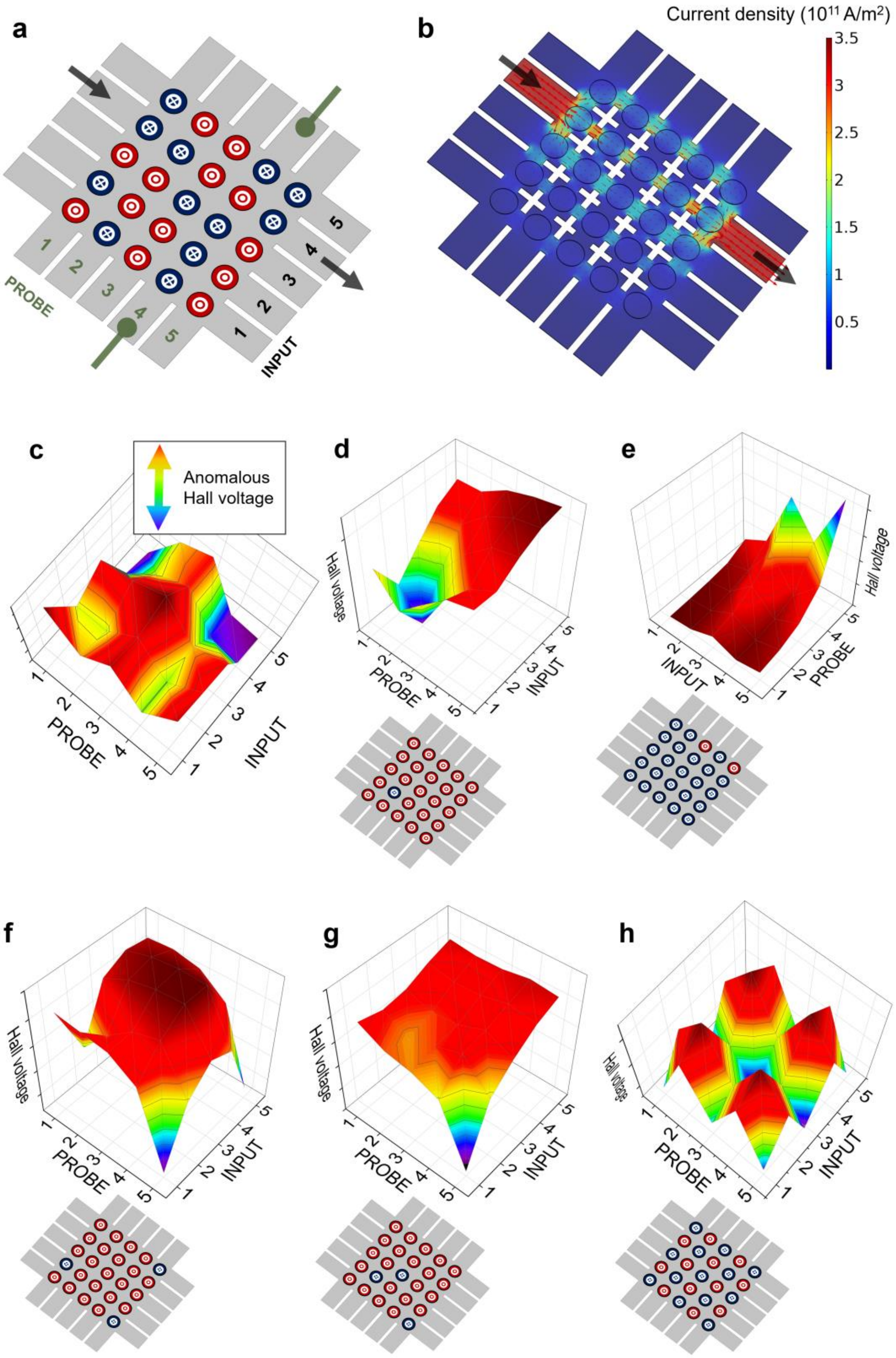

Figure S10. Local readout with planar electrodes. (**a**) Schematic of the electrodes and the readout scheme. Red (blue) circles represent nanomagnets with "up" ("down") magnetization. (**b**) An example of a non-uniform current distribution in the system. (**c**) Readout pattern produced by the magnetic state shown in (a). (**d**)-(**h**) Readout patterns corresponding to

different magnetic states indicated to the bottom left of the plots. The increment of the vertical axis is 2 mV in all cases.

Inhomogeneities in the current density distribution in the Hall bar used to measure the magnetic state of the 2D Ising systems may result in different currents flowing through the different nanomagnets, resulting in unequal contributions to R_{Hall}. This may lead to a distortion in the one-to-one correspondence between R_{Hall} and m_{avg}. This effect is negligible for lattices of limited size placed on a symmetric Hall bar, as can be seen from the 25 rather homogenous steps in R_{Hall}, corresponding to single nanomagnets switching, in Figure S9a. However, the inhomogeneity in the current density can be deliberately increased by adding more electrodes to the system, and this can be used to extract individual states of the nanomagnets and not just m_{avg}. To demonstrate this, we performed COMSOL simulations of a 5x5 lattice with 5 electrodes on each side, organized into 5 pairs of “input” electrodes for applying current and 5 pairs of “probe” electrodes for measuring the resultant anomalous Hall voltage (Figure S10a). Changes in current distribution, which depend on which electrodes are used, result in the probing of different areas of the lattice. For example, applying a voltage to the 3rd pair of input electrodes results in the current density distribution shown in Figure S10b.

We then applied a test sequence, which consisted of applying a probing current to a pair of input electrodes while reading out Hall voltage from one of the pairs of transverse probe electrodes. This was repeated for all the combinations of input and probe electrode pairs, giving a matrix of 25 measurements. A representation of this readout is shown in Figure S10c, where *x* and *y* coordinates are input and probe pair number, and the *z* (vertical) coordinate is the corresponding anomalous Hall voltage readout. The readout in Figure S10c corresponds to the magnetic state of Figure S10a. Nanomagnets that are magnetized “up” (“down”) produce a positive (negative) contribution to the readout, as one can see by comparing Figure S10a and Figure S10c, particularly in the corners of the lattice. However, there is no one-to-one correspondence between the magnetic state and the electrical readout because every nanomagnet contributes to the Hall voltage for each of the 25 input-probe electrode combinations. Nevertheless, the magnetic state of the lattice is clear from the readout pattern in some simple cases such as those shown in Figures S10d, e and f, in which peaks or troughs correspond to the few nanomagnets magnetized in the opposite direction to the majority of the lattice. The more complex magnetic states are difficult to understand from the readout pattern (e.g. in Figure S10g; in Figure S10h some peaks correspond to nanomagnets with “down” magnetization due to the contribution of the surrounding nanomagnets).

To understand the Hall voltage readout for the general case of arbitrary magnetic states, we simulated readout patterns where only one single nanomagnet has a magnetization orientation that differs from the others, which we call “special readout patterns”. There are 25 such special readout patterns, and one example is shown in Figure S10d. For the general case, any Hall voltage readout, such as those illustrated in Figures S10c to S10h, can be constructed by summing these 25 special readout patterns weighted by a +1 or -1 coefficient, which reflects the orientation of the the single nanomagnet with differing magnetization orientation in the array. There is not always a direct correlation between the peaks and troughs of the general readout patterns, and the “up” or “down” states of nanomagnets, as can be seen in Figure S10h. However, for certain nanomagnets, the sign of the Hall voltage is always correlated with their magnetic state. This is true for the nanomagnets near the input and probe electrodes, which contribute most significantly to the readout and thus do not get masked by contributions from their neighbours.

Identifying such cases allows us to remove their contributions with the correct signs from the general readout, and then to proceed with the next-largest contributors. We describe this in more detail as follows:

- The nanomagnets in the corners of the lattice provide the largest contributions to the readout, overriding any signal from the rest of the system. The sign of the readout for the combination of inputs 1 and 5 with probes 1 and 5 thus corresponds to the magnetic states of these nanomagnets. Using this, we determine their magnetic states ("up" or "down") and subtract these special readouts from the initial readout pattern with the corresponding (+1 or -1) signs.
- Once the contributions of the nanomagnets in the corners have been subtracted, the next-largest nanomagnet contributors are the sets of three nanomagnets between the pairs of corner nanomagnets (e.g., probe 1 in combination with inputs 2, 3, 4, or input 1 in combination with probes 2, 3, 4) since they are the closest to the electrodes among the remaining contributors. The magnetic states of these nanomagnets can thus be determined from the sign of the corresponding readout and then their corresponding special readouts can be subtracted accordingly.
- After these contributions have been subtracted, the eight nanomagnets forming a square around the central magnet, followed by the central one, can be read out and subtracted.

Peeling off the readout in this way provides a route to retrieve all of the states of the 25 individual nanomagnets from the electrical readout of any magnetic state. This means that any parameter of the system, such as q_{NN}, q_{2NN} and q_{3NN}, can be determined using the planar electrodes. The temporal resolution of such measurements is limited by the time necessary to perform the 25 measurements with sufficient signal-to-noise ratio. Therefore, while m_{avg} can be tracked with a temporal resolution limited only by the readout circuitry, measurements of q_{NN}, q_{2NN} and q_{3NN} may be slower. Electrical setups such as that described in Ref. 7 can be used to achieve ns temporal resolution. We note that our approach allows one to distinguish between symmetrically placed nanomagnets unlike in previous works[10,11].

From a computational perspective, these results are important because they show that, even if individual magnetic states are not read out directly (e.g., when using a crossbar), they can still be retrieved by performing a set of linear operations. In the context of reservoir computing, this means that the use of this readout approach with multiple electrodes can be computationally equivalent to knowing the exact magnetic configuration of the system.

Finally, it is worth mentioning that the maximum system size, to which this local electrical readout method with planar electrodes can be applied to determine the magnetic state, may be limited by the lower signal from central areas that are far from any electrodes. Maximizing the current density, for example, by adding cutouts in the electrode as shown in Figure S10b, may be helpful in alleviating this problem. In addition, using machine learning instead of the sequential "peeling off" of the readout discussed here may also be helpful in extending this method to lattices of larger sizes or more complex geometries.

Supplementary Note 8: Transformation improvements and Mackey-Glass prediction

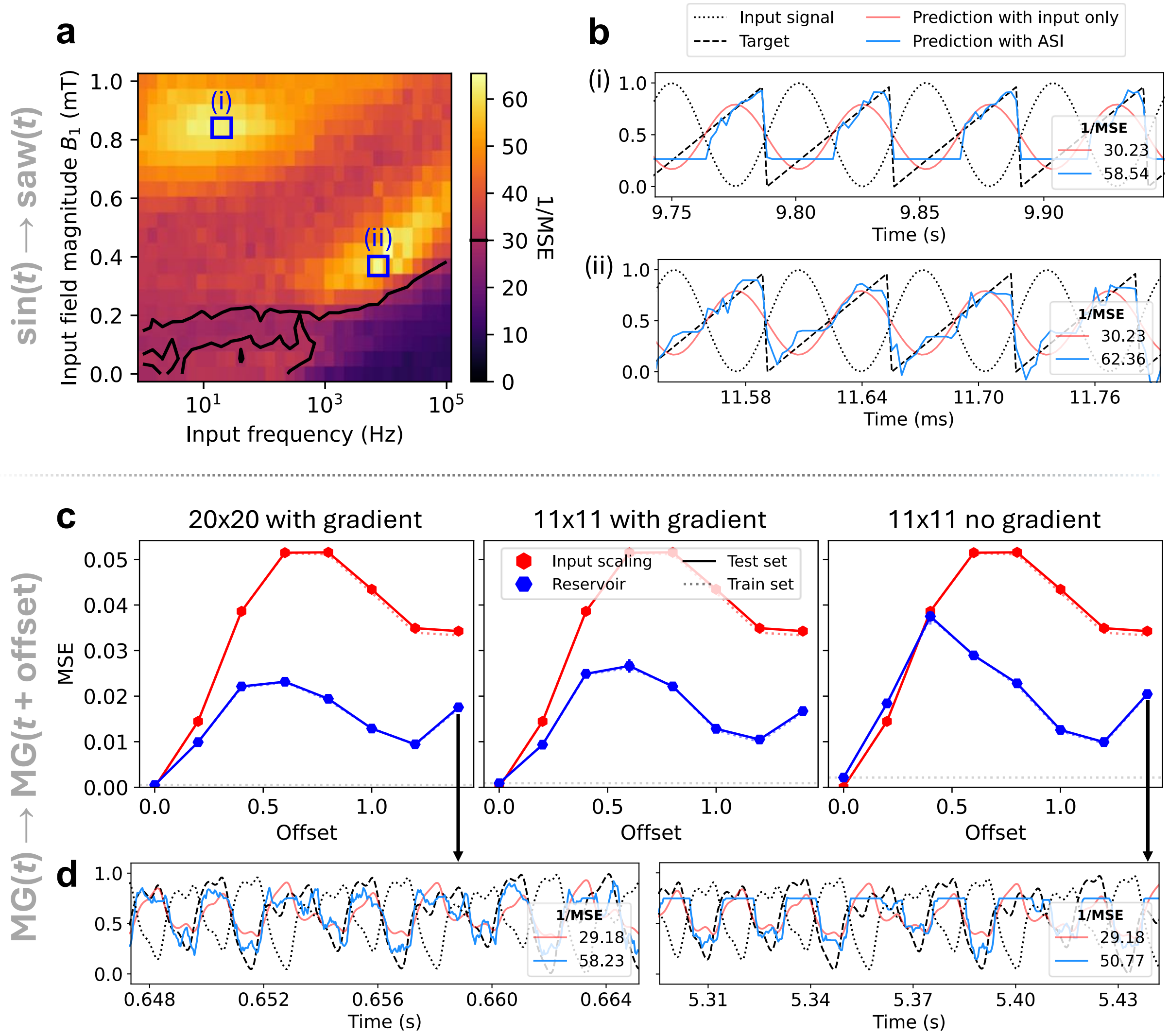

Figure S11. Signal transformation of different sized lattices with and without a gradient in E_{EA} and magnetic moment. The parameters E_{MC} = 2.5k_BT and E_{EA} = 20k_BT ± 5% are employed for all figure parts. (**a**) Inverse MSE for a sine wave-to-sawtooth transformation for a 20×20 lattice with a 10% gradient in E_{EA} and magnetization, as a function of input field frequency and maximum input field B_1. The minimum input field B_0 = -0.2 mT. (**b**) Temporal trace of the sine wave-to-sawtooth transformation for the two hotspots in (a). The input signal is given by black dotted lines and the target is given by black dashes. The prediction with (without) the reservoir is given by blue (red) lines. (**c**) Performance for a Mackey-Glass prediction given by MSE as a function of desired future time 'offset', for two system sizes with and without a gradient. (**d**) Temporal traces of the Mackey-Glass prediction with offset 1.4, both for the 20×20 lattice with a gradient and for the 11×11 lattice without a gradient.

The sine wave-to-sawtooth transformation presented in the main text was simulated for an 11×11 lattice of spins that is just like the experimental system of nanomagnets. However, several modifications to the lattice can improve performance. Firstly, enlarging the system is beneficial because this reduces thermal noise in the output, as each readout value becomes the average of more magnets. Secondly, introducing a gradient in effective anisotropy E_{EA} and magnetic moment

of the spins to obtain a greater variation in the readout values will also yield a better transformation. Here, we implement a gradient where E_{EA} and magnetic moment of the spins gradually gets larger going from one side of the lattice to the other. This gradient in E_{EA} means that magnets on one side of the lattice relax faster than on the other side, providing several different short-term memory timescales. A "gradient of *10*%" means that the E_{EA} on the right (left) side of the lattice is *10*% higher (lower) than the average. The exchange coupling *J* between the nanomagnets is set to 0.

The effect of extending the lattice and adding a gradient in E_{EA} and magnetic moment is presented in Figure S11. Figures S11a and S11b concern a sine wave-to-sawtooth transformation (as in the main text), but for a larger 20×20 system with a gradient of 10%. Note that, while a low MSE (or high 1/MSE) is often indicative of a good transformation, this is not necessarily the case in noisy systems where the noise imposes a lower bound to the MSE. For example, in Figure S11a, there exist two distinct combinations of input magnitude and frequency that both give a good MSE (blue boxes), yet the temporal traces (Figure S11b) reveal that the higher-frequency input (7.5 kHz / 0.37 mT) yields a much better sawtooth than the lower-frequency input (20 Hz / 0.84 mT). For the low-frequency input (upper panel), the magnetic state saturates at large amplitudes of the sine input, so that the low peak in the sawtooth is cut off. Nevertheless, the prediction follows the steep drop of the sawtooth better than with the high-frequency input (due to a finite relaxation time), thus giving similar MSE. This illustrates that, not only MSE should be considered as a metric for a high-performance reservoir, but that the frequency adjustment is also key for obtaining a correspondence in shape between the reservoir-based prediction and the target.

When the size of the nanomagnet array is increased, thermal noise in the output signal will be averaged over more nanomagnets, thus giving a reduction in the random error of the output (affecting reproducibility) but not the systematic error (the ability of the system to transform the signal). Thus, for an infinitely large system, the output signal would be perfectly reproducible, but not necessarily equal to the target function.

In Figures S11c and S11d, we explicitly compare the results from large and small systems, with and without a gradient in E_{EA} and magnetic moment, but now for a time series prediction task. We use the standard task of a chaotic Mackey-Glass (MG) oscillator[12] whose states must be predicted at a given amount of time in the future, which is referred to as the 'offset'. The following parameters of MG equation were used: τ = 23.0, β = 0.2, γ = 0.1, n = 10.0 (Ref. [13]). The MSE of the various lattices as a function of MG offset, i.e., how far into the future should be predicted, is shown in Figure S11c. The datapoints show the best MSE among all lattices of a given class for a given offset. For instance, for offset = 1 of the panel "20x20 with gradient" in Figure S11c, we tested the prediction task on 20×20 lattices with various gradient strengths while applying inputs with different frequencies *f* and encoding fields B_0 and B_1, and then selected the best MSE among the results. The effect of adding a gradient in both E_{EA} and the magnetic moment, and enlarging the system turn out to be significant. This improvement can be seen when comparing the temporal views in Figure S11d, where the larger system with a gradient (lefthand panel) produces a closer prediction than the smaller system without a gradient (righthand panel). For example, for the smaller system, it can be seen that, for the prediction with artificial spin lattice (blue curve), the top of the peaks is cut off. This is not the case for the larger system. These results are similar to the performance of other magnetic reservoirs[14–16].

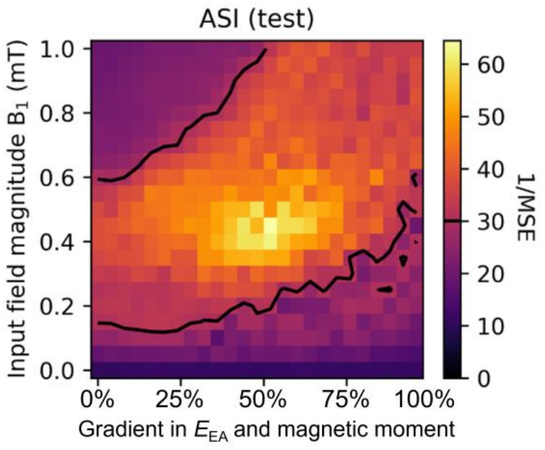

Figure S12. Dependence of the system performance (1/Mean Squared Error) on the relative gradient of effective anisotropy E_{EA} and magnetic moment of the spins, and input field magnitude B_1.

To assess the optimal value for the gradient, we estimated the artificial spin lattice's performance when carrying out the sine wave-to-sawtooth transformation as a function of the gradient (Figure S12). A gradient of 50% leads to the smallest MSE. This result provides guidance for the degree of non-homogeneity optimal in these lattices when applied to transformation tasks in reservoir computing.

Finally, we note that the optimal input frequency can be controlled by the anisotropy E_{EA} of the nanomagnets. The optimal frequency of the system shown in Figure 6 of the main text is 200Hz. This is for an array of nanomagnets with an anisotropy of $E_{EA} = 20k_BT$. Using the exponential Néel-Arrhenius law, one would arrive at an optimal frequency of $200\times e^{-40}$ Hz for nanomagnets with $E_{EA} = 60k_BT$ and an optimal frequency of $200\times e^{10}$ or ~4.4 GHz for nanomagnets with $E_{EA} = 10k_BT$. The lattices are therefore sensitive to the anisotropy of the nanomagnets, while their maximum optimal frequency is limited only by the timescale of the ferromagnetic dynamics.

Supplementary Note 9: Experimental lowering of the switching energy with current-induced Joule heating and spin-orbit torques

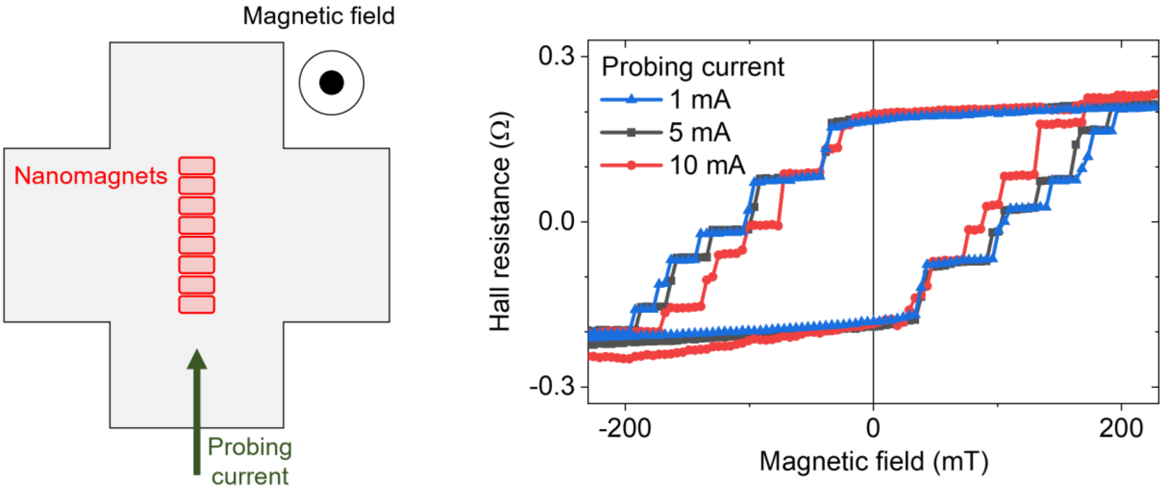

Figure S13. Hall resistance versus out-of-plane magnetic field for different probing currents.

Applying Joule heating and spin current to the lattice of nanomagnets with perpendicular magnetic anisotropy can lower the energy barrier to switching of the nanomagnets. Here we look at the effect of Joule heating and spin current on a 1D chain of 8 nanomagnets, similar to that shown in Figure S15, but which does not show spontaneous ordering at room temperature at the experimental timescale. The lattice is placed on a Ta/Pt Hall bar. The current is used for probing and simultaneously acts as a source of Joule heating and spin-orbit torque, and we find that an increase in current leads to switching of the nanomagnets at smaller fields (Figure S13). Since neither Joule heating nor spin-orbit torques break the symmetry between the “up” and “down” states, the energy barrier is modified equally for all nanomagnets, and the order in which the nanomagnets switch is governed by the magnetostatic coupling. Therefore, we imagine that this approach could be suitable for annealing artificial spin lattices with perpendicular anisotropy, without the need to apply a magnetic field or heat.

Supplementary Note 10: Magnetostatic coupling enhancement with a Permalloy underlayer

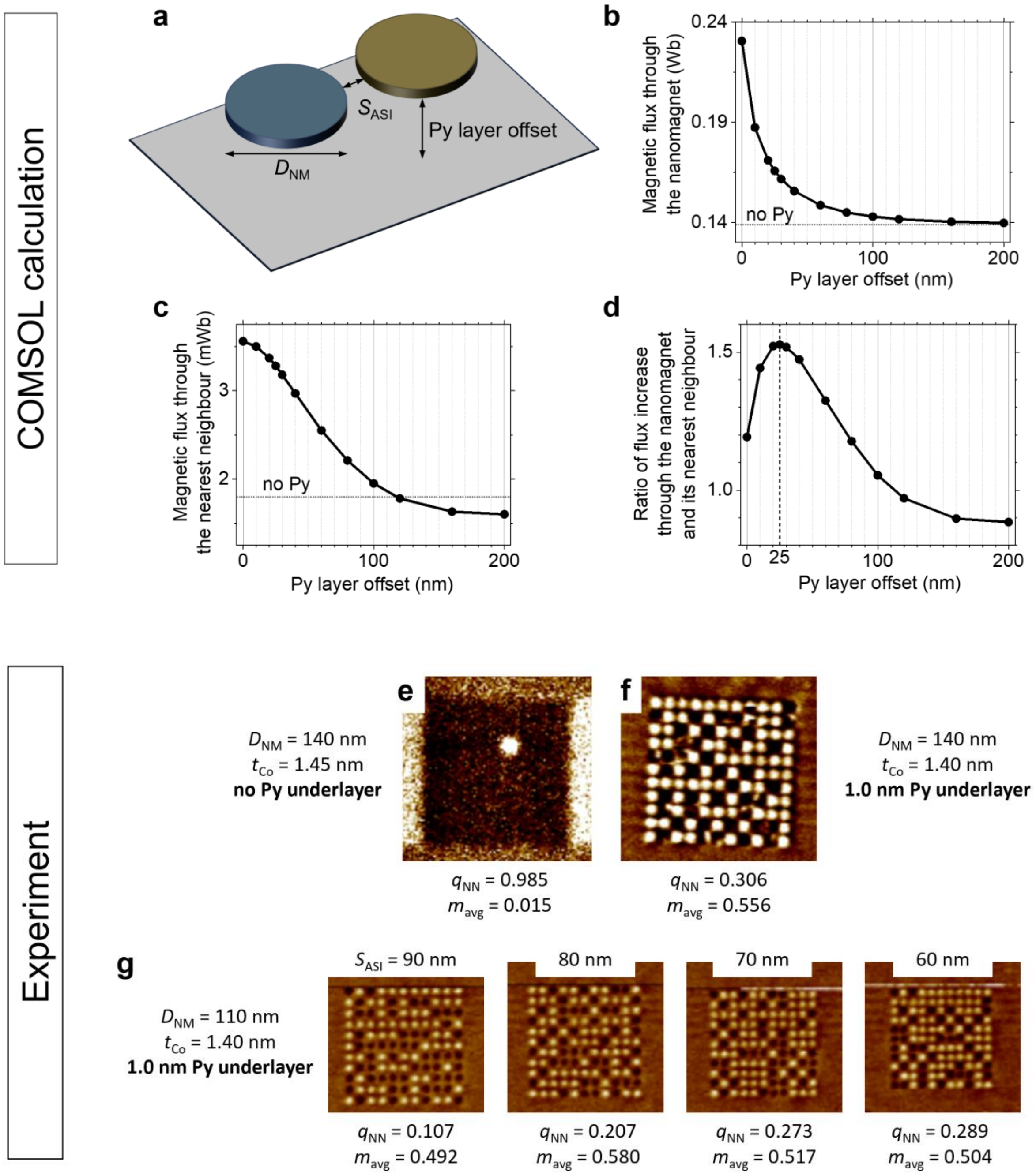

Figure S14. Enhancement of intermagnet coupling and spontaneous ordering through mediation of the magnetic flux with a permalloy underlayer. All images were taken at $t \sim 1000$ s. (**a**) Schematic of the simulation. (**b**) Change of magnetic flux through the nanomagnet itself with the distance to the Py underlayer. (**c**) Change of magnetic flux through the neighbouring nanomagnet with the distance to the Py underlayer. (**d**) Ratio between (c) and (b). (**e**) and (**f**) Spontaneous switching in 11×11 lattices in similar multilayer stacks without and with Py underlayer. The Py offset is 25 nm. (**g**) Spontaneous switching in an 11×11 lattice for nanomagnet diameter D_{NM} = 110 nm and different separations S_{ASI}. The distance to the Py layer is 25 nm.

It is known that a Permalloy (Py) underlayer can increase the magnetostatic coupling in lattices of nanomagnets with perpendicular magnetic anisotropy that do not show spontaneous ordering at experimental timescales[17]. The magnetostatic interactions in these systems are weak enough to prevent the individual nanomagnets from forming multidomain states but are also too weak to give spontaneous ordering. Thus, both the lack of rapid ordering among the nanomagnets and their tendency to remain in single-domain states are driven by the same principle of demagnetization field energy minimization. For example, increasing D_{NM} provides stronger coupling but also, above a certain diameter, results in the undesirable formation of multidomain states. Thus, it is critical to increase the coupling energy in such a way that the demagnetization energy does not result in a multidomain state. To address this, we performed COMSOL calculations to determine how a Py underlayer would change the magnetic flux in a nanomagnet as well as through its neighbour. The purpose was to find parameters at which the magnetic flux through the nanomagnet itself is increased less significantly than the flux through its neighbour, thus promoting magnetostatic coupling more than formation of a multidomain state.

For the calculations, a saturation magnetization of 575 kA/m and 1063 kA/m was used for Py and Co, respectively. The magnetization was fixed to be in-plane for Py and out-of-plane for Co nanomagnets. The pair of circular Co nanomagnets were separated by 1 nm. The surroundings of the magnets were given the magnetic permeability of vacuum.

The dependence of the magnetic flux in the nanomagnet and a neighbouring nanomagnet (for a pair of coupled nanomagnets – see schematic in Figure S14a) on the vertical separation of the Py layer from the bottom of the nanomagnets ("Py layer offset") is shown in Figures S14b and c. The ratio between the trends (Figure S14d) indicates the Py layer offset where the flux through the neighbour is larger than that through the original nanomagnet. The best ratio of ~1.6 is achieved at a Py layer offset of 25 nm.

Using this as a guide, we fabricated lattices with N_{Co} = 7, t_{Co} = 1.40 nm and nanomagnet diameters D_{NM} of 110, 140 and 170 nm on top of Py ($Ni_{80}Fe_{20}$) layers with thicknesses ranging from 0 to 7 nm and separated from the lattice by 25 nm. We only show the results for a Py thickness of 1 nm since this gave a noticeably better coupling enhancement than for the other thicknesses. The lattices were measured with MFM following the same procedure as described in the main text. At D_{NM} = 170 nm, the majority of the nanomagnets were in a multidomain state, as expected due to the increased flux through the nanomagnet itself. The MFM measurement for D_{NM} = 140 nm is shown in Figure S14f. Here, we observe a significant increase in spontaneous ordering compared to the system with no permalloy underlayer despite the larger t_{Co} (1.45 nm compared with 1.40 nm) of the latter (Figure S14e). Some multidomain states can also be seen in the nanomagnets in Figure S14f. Notably, significant spontaneous relaxation is also observed in the system with D_{NM} = 110 nm, even for S_{ASI} = 90 nm (Figure S14g). At the same time, no multidomain states can be seen for these parameters.

These results show that enhancement of the intermagnet coupling by adding an underlayer can be significant enough to modify a system that is frozen on experimental timescales to one that relaxes to a low energy state. Nevertheless, special care must be taken to prevent the formation of multidomain states, which means that lattices with smaller D_{NM} might benefit more from this approach.

Supplementary Note 11: Modification of E_{MC} by altering the nanomagnet shape

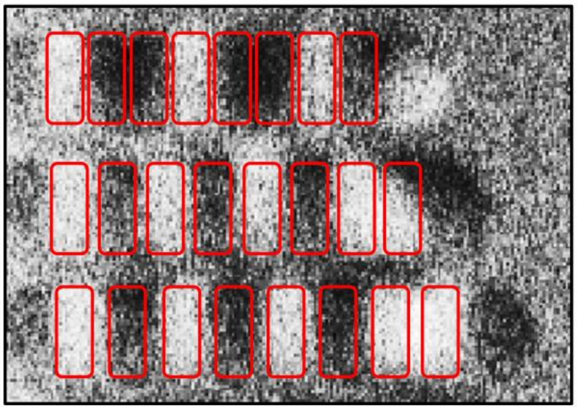

Figure S15. Chains of 112.5 nm × 49.5 nm nanomagnets with perpendicular magnetic anisotropy. The separations between the nanomagnets in the three rows, going from top to bottom, are 60, 50 and 40 nm.

Changing the geometry of a lattice and its nanomagnets can bring the “magnetic centres of mass” of the nanomagnets closer together and thus increase E_{MC}. To demonstrate this, we performed an MFM measurement of 1D chains of rectangular nanomagnets with rounded corners (Figure S15). While the ordering dynamics in such a system are not the same as in a 2D rectangular lattice, the degree of spontaneous ordering achieved at t ~ 1000 s for a given nanomagnet size can serve as a useful reference for the strength of the interaction between the nanomagnets. Here the same measurement protocol as in the main text was used. Almost perfect order is observed for a stack with N_{Co} = 7, t_{Co} = 1.4 nm despite separations of up to 60 nm and a much smaller nanomagnet area of ~5400 nm^2 compared with an area of ~22700 nm^2 for circular nanomagnets with D_{NM} = 170 nm.

Such an approach could not only provide a way to enhance E_{MC}, as shown here, but also a method to tune the coupling strengths between different neighbours independently, by changing relative lengths of the borders between them. This fabrication of an array of nanomagnets with a precisely engineered spatially-varying intermagnet coupling would enable the creation of artificial spin lattices with novel emergent properties.

References

1. Maes, J. *et al.* The design, verification, and applications of Hotspice: a Monte Carlo simulator for artificial spin ice. Comp. Phys. Commun. 313, 109643 (2025).
2. Brown, W. F. Thermal Fluctuations of a Single-Domain Particle. *Phys. Rev.* **130**, 1677–1686 (1963).
3. Dmytriiev, O., Meitzler, T., Bankowski, E., Slavin, A. & Tiberkevich, V. Spin wave excitations of a magnetic pillar with dipolar coupling between the layers. *J. Phys.: Condens. Matter* **22**, 136001 (2010).
4. Joseph, R. I. Ballistic demagnetizing factor in uniformly magnetized cylinders. *J. Appl. Phys.* **37**, 4639–4643 (1966).
5. Politi, P. & Pini, M. G. Dipolar interaction between two-dimensional magnetic particles. *Phys. Rev. B* **66**, 214414 (2002).
6. Hu, W. *et al.* Distinguishing artificial spin ice states using magnetoresistance effect for neuromorphic computing. *Nat. Commun.* **14**, 2562 (2023).
7. Sala, G. *et al.* Real-time Hall-effect detection of current-induced magnetization dynamics in ferrimagnets. *Nat. Commun.* **12**, 656 (2021).
8. Takahashi, Y. *et al.* Spin-orbit torque-induced switching of in-plane magnetized elliptic nanodot arrays with various easy-axis directions measured by differential planar Hall resistance. *Appl. Phys. Lett.* **114**, 012410 (2019).
9. Branford, W. R., Ladak, S., Read, D. E., Zeissler, K. & Cohen, L. F. Emerging Chirality in Artificial Spin Ice. *Science* **335**, 1597–1600 (2012).
10. Neumann, A. *et al.* Influence of long-range interactions on the switching behavior of particles in an array of ferromagnetic nanostructures. *New J. Phys.* **16**, 083012 (2014).
11. Bhowmik, D., You, L. & Salahuddin, S. Spin hall effect clocking of nanomagnetic logic without a magnetic field. *Nat. Nanotechnol.* **9**, 59–63 (2014).
12. Mackey, M. C. & Glass, L. Oscillation and Chaos in Physiological Control Systems. *Science* **197**, 287–289 (1977).
13. Grassberger, P. & Procaccia, I. Measuring the strangeness of strange attractors. *Physica D* **9**, 189–208 (1983).
14. Lee, O. *et al.* Task-adaptive physical reservoir computing. *Nat. Mater.* **23**, 79–87 (2024).
15. Gartside, J. C. *et al.* Reconfigurable training and reservoir computing in an artificial spin-vortex ice via spin-wave fingerprinting. *Nat. Nanotechnol.* **17**, 460–469 (2022).
16. Stenning, K. D. *et al.* Adaptive Programmable Networks for In Materia Neuromorphic Computing. Preprint at https://doi.org/10.21203/rs.3.rs-2264132/v1 (2022).
17. Kempinger, S. *et al.* Field-Tunable Interactions and Frustration in Underlayer-Mediated Artificial Spin Ice. *Phys. Rev. Lett.* **127** (2021).
